# Advancements and Strategies in CsPbI_2_Br Perovskite Solar Cells for Enhanced Efficiency and Stability

**DOI:** 10.3390/nano15070483

**Published:** 2025-03-24

**Authors:** Fanbei Sun, Tingting Hou, Kexuan Xie, Xinghua Zhu, Dingyu Yang, Xin Liu

**Affiliations:** 1 Sichuan Province Key Laboratory of Optoelectronic Sensor Devices and Systems, Sichuan Meteorological Optoelectronic Sensor Technology and Application Engineering Research Center, Chengdu IC Valley Industrial College, College of Optoelectronic Engineering, Chengdu University of Information Technology, Chengdu 610225, China; fanbei_sun@139.com (F.S.); 18782114977@139.com (T.H.); 1112284584@139.com (K.X.); 2 School of Materials Science and Engineering, Xihua University, Chengdu 610039, China; 3 Dazhou Industrial Technology Research Institute, Dazhou 635000, China; 4 Intelligent Manufacturing Industry Technology Research Institute, Sichuan University of Arts and Science, Dazhou 635000, China

**Keywords:** CsPbI_2_Br perovskite solar cells, film deposition techniques, interface engineering, charge transport layers, high performance

## Abstract

In recent years, inorganic perovskite solar cells (IPSCs), especially those based on CsPbI_2_Br, have attracted considerable attention owing to their exceptional thermal stability and a well-balanced combination of light absorption and phase stability. This review provides an extensive overview of the latest progress in CsPbI_2_Br PSCs, focusing on film deposition techniques, crystallization control, interface engineering, and charge transport layers (CTLs). High-efficiency CsPbI_2_Br PSCs can be achieved through the optimization of these key aspects. Various strategies, such as solvent engineering, component/additive engineering, and interface optimization, have been explored to enhance the quality of CsPbI_2_Br films and improve device performance. Despite significant progress, challenges remain, including the need for even higher quality films, a deeper understanding of interface energetics, and the exploration of novel CTLs. Additionally, long-term stability continues to be a critical concern. Future research should focus on refining film preparation methods, developing sophisticated interfacial layers, exploring compatible charge transport materials, and ensuring device durability through encapsulation and moisture-resistant materials.

## 1. Introduction

In renewable energy technologies, PSCs have emerged as a formidable contender for the next generation of photovoltaic (PV) devices. Their exceptional optoelectronic characteristics, including high absorption coefficients across the visible spectrum, tunable bandgaps, extended carrier diffusion lengths, and low exciton binding energies, make them an attractive alternative to traditional silicon-based solar cells [1,2,3,4]. To date, the power conversion efficiency (PCE) of PSCs has achieved 27.0%, making it comparable with current commercial PV technologies [5,6,7,8,9]. However, the organic components in halide perovskites, such as those containing methylammonium (MA^+^) or formamidinium (FA^+^), limit the commercialization of these materials due to their inadequate thermal and chemical stability. For example, the MAPbI_3_ perovskite material, which has a bandgap (E_g_) of 1.51 eV, exhibits thermodynamic instability and experiences a phase transition from a tetragonal to a cubic configuration at a temperature of 57 °C [10]. By substituting the MA^+^ ion with the FA^+^ ion, the E_g_ of FAPbI_3_ decreases to 1.48 eV, leading to a significant enhancement in the thermal stability of the FAPbI_3_ film [11]. However, at ambient temperature, FAPbI_3_ exhibits a non-perovskite hexagonal yellow δ phase, which substantially impacts the performance of solar cells [12]. Consequently, it has been suggested to use Cs^+^ ions to replace both MA^+^ and FA^+^, as the fully inorganic CsPbX_3_ (X = I or Br) perovskite maintains its stability up to 460 °C [13,14]. Typically, CsPbI_3_ (E_g_ = 1.73 eV) requires sintering at a high temperature of 300 °C to form a perovskite-type black cubic α-phase structure. Upon cooling to room temperature, it degrades into a non-perovskite orthorhombic yellow δ-phase structure (E_g_ = 2.82 eV) [15]. Alternatively, the E_g_ of CsPbBr_3_ and CsPbIBr_2_ are approximately 2.32 eV [16] and 2.05 eV [17], respectively. These values are excessively high, making these materials unsuitable for serving as absorber layers in PSCs.

Among the diverse perovskite compositions, inorganic CsPbI_2_Br has distinguished itself due to its enhanced thermal and phase stability, which are critical for long-term operational reliability in real-world applications [14]. The incorporation of bromide ions into the CsPbI_3_ lattice to form CsPbI_2_Br not only stabilizes the black perovskite phase but also adjusts the Goldschmidt tolerance factor to a more favorable range, ensuring structural stability. This substitution leads to an E_g_ of 1.8–1.9 eV, which has shown great potential in the field of multi-junction cascaded and semi-transparent solar cells [18,19,20,21,22,23].

Despite the inherent advantages of CsPbI_2_Br, there are still many challenges to realizing its full potential in PV applications. One of the primary hurdles is achieving high-quality perovskite films with optimal morphology. Poor film quality, characterized by pinholes, small grain sizes, and rough surfaces, can significantly impede charge transport and increase recombination losses, thereby reducing the efficiency of the device. To overcome these challenges, researchers have explored various strategies aimed at optimizing the crystallization process of CsPbI_2_Br films. Solvent engineering, for instance, involves carefully selecting solvents and their ratios to control the solubility and crystallization rate of the perovskite precursor [24]. Antisolvent treatments, such as dripping a miscible solvent onto the wet film during spin coating, have been found to promote rapid crystallization and the formation of larger grains [25]. Furthermore, additive engineering, where small amounts of additives are introduced into the precursor solution, can modify the crystallization kinetics and reduce defects within the film [26,27]. Another crucial aspect of enhancing PSC performance is interface engineering. The interfaces between the CsPbI_2_Br layer and the CTLs play a pivotal role in determining the efficiency of charge extraction and transport [18,28]. Energy offsets at these interfaces can create barriers to charge transfer, leading to increased recombination and a reduced photocurrent. To mitigate these effects, interfacial layers with appropriate energy level alignments and high conductivity are introduced. These layers act as bridges, facilitating efficient charge transfer and minimizing energy losses.

In addition to optimizing the perovskite film and interfaces, developing novel CTLs is essential for further improving PSC performance [29,30,31]. Traditional CTLs, such as TiO_2_ and Spiro-OMeTAD, while demonstrating acceptable performance, may not fully satisfy the stringent requirements of CsPbI_2_Br with respect to energy level alignment and long-term stability. Researchers have therefore explored alternative materials, including two-dimensional materials such as graphene and transition metal dichalcogenides, metal oxides such as SnO_2_ and NiO_x_, and organic polymers. These materials offer improved electrical and optical properties, better energy level alignment, and enhanced stability, contributing to device performance and lifetime [29,32].

The relentless pursuit of efficiency and stability improvements has led to significant progress in CsPbI_2_Br PSCs. Current devices have achieved PCEs exceeding 18%, a testament to the effectiveness of the aforementioned strategies [33,34,35]. However, to reach the commercialization threshold and compete with established PV technologies, further advancements are necessary. Future research should focus on several key areas. Firstly, continued efforts to refine film preparation methods and explore new additives are crucial for achieving even higher quality perovskite films. Secondly, a deeper understanding of interface energetics and the development of more sophisticated interfacial layers will help minimize energy barriers and reduce recombination losses. Thirdly, the exploration of novel CTLs with superior properties and compatibility with CsPbI_2_Br is essential for pushing the boundaries of PSC performance. Moreover, long-term stability remains a paramount concern for any PV technology. Research into encapsulation techniques, moisture-resistant materials, and device architectures that can withstand harsh environmental conditions is vital for ensuring the durability of CsPbI_2_Br PSCs.

**Figure 1:**
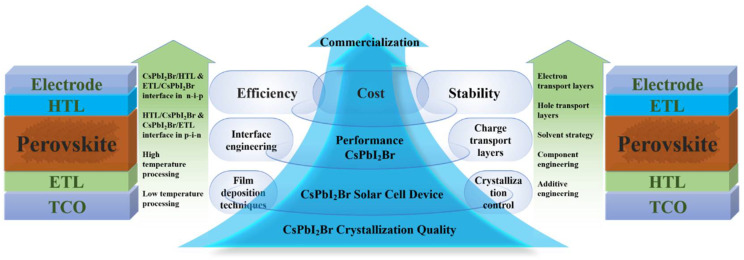
Summary of approaches for enhancing the performance of CsPbI_2_Br PSCs.

In this review, we illuminate the latest progress in mixed-halide CsPbI_2_Br perovskite materials tailored for PV applications. High-efficiency CsPbI_2_Br PSCs can be meticulously crafted through the adoption of several potent strategies, including the refinement of film deposition techniques, precise crystallization control, meticulous interface engineering, and the incorporation of optimized CTLs (as depicted in Figure 1). By capitalizing on the combined benefits of these methodologies, substantial enhancements in the stability and efficiency of CsPbI_2_Br PSCs can be realized. Furthermore, we delve deeply into the fundamental mechanisms underlying how crystallization processes and interface engineering directly impact the formation of superior-quality CsPbI_2_Br films, which, in turn, significantly influence the performance of the resulting PV devices. We underscore the crucial role these factors play in determining the efficiency of the devices. Lastly, we venture into exploring and discussing promising pathways for further elevating the PV performance of these devices and propelling the commercialization of IPSCs.

## 2. CsPbI_2_Br Film Deposition Techniques

### 2.1. High-Temperature Processing

High-temperature annealing serves as a pivotal technique for producing phase-pure, stable CsPbI_2_Br perovskite films with superior optoelectronic properties. The cubic α phase of CsPbI_2_Br, which is essential for efficient light absorption and charge transport, generally necessitates elevated temperatures (>200 °C) to overcome kinetic barriers and inhibit the formation of non-perovskite δ phases. Nevertheless, high-temperature processing presents significant challenges in terms of scalable manufacturing and compatibility with flexible or tandem device architectures. This section investigates strategies to achieve an optimal balance between thermal stability, grain growth, and interfacial quality under high-temperature conditions, with an emphasis on refining annealing protocols, precursor interactions, and phase evolution mechanisms.

**Figure 2:**
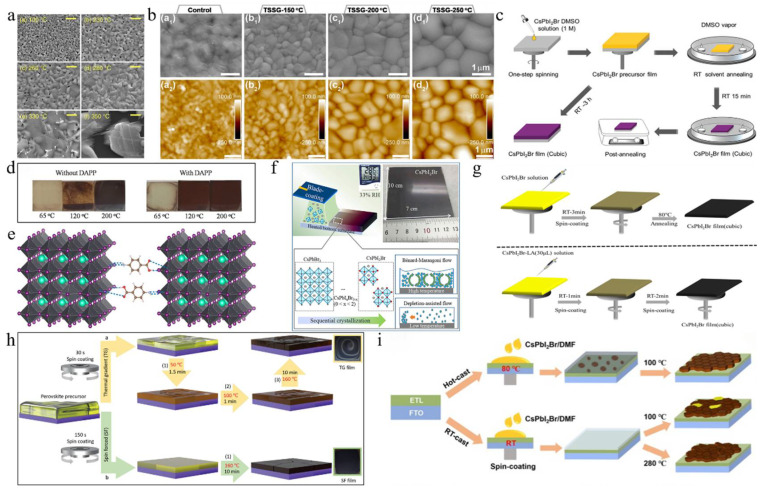
(**a**) SEM images of CsPbI_2_Br films annealed at different temperatures. Reprint with permission [36]. Copyright 2017, American Chemical Society. (**b**) Morphology characterization of the control sample and TSSG CsPbI_2_Br films. Reprint with permission [37]. Copyright 2021, American Chemical Society. (**c**) Schematic diagram of preparation procedures of the CsPbI_2_Br films with RT DMSO vapor annealing and direct thermal annealing, respectively. Reprint with permission [40]. Copyright 2018, Wiley-VCH. (**d**) Comparison of the air stability (humidity: ≈30%) of the CsPbI_2_Br perovskite films without and with DAPP annealed at different temperatures. Reprint with permission [41]. Copyright 2018, Wiley-VCH. (**e**) Schematic illustration of two neighboring grain structures cross-linked by ABA. Reproduced with permission [42]. Copyright 2018, American Chemical Society. (**f**) Schematic illustration of ambient blading. Reproduced with permission [43]. Copyright 2019, Elsevier. (**g**) Schematic diagram of the preparation of cubic-phase perovskite at RT and at 80 °C. Reproduced with permission [44]. Copyright 2020, Elsevier. (**h**) Schematic illustration of depositing the perovskite films by TG (conventional) and SF methods. Reprint with permission [45]. Copyright 2022, Wiley-VCH. (**i**) Schematic diagram of CsPbI_2_Br films prepared by conventional RT-casting (**down**) and hot-casting (**top**) processes, respectively. Reproduced with permission [46]. Copyright 2019, Elsevier.

To characterize the morphological evolution, Park et al. annealed CsPbI_2_Br, prepared by one-step spin coating, at temperatures from 100 °C to 350 °C (Figure 2a) [36]. The crystal size increased with temperature, following the Ostwald ripening mechanism. Notably, the film annealed at 280 °C showed a pinhole-free, uniform, and dense microstructure, indicating superior performance and enhanced phase stability in humid conditions. The top-seeded solution growth (TSSG) technique was used to anneal the CsPbI_2_Br film at 150 °C, 200 °C, and 250 °C (Figure 2b) [37]. Results showed that increasing annealing temperature enlarged grain size, reduced crystal defects, and improved thermal stability. PSCs fabricated at 200 °C achieved the highest PCE of 14.84%. Due to the incorporation of iodine ions during annealing, CsPbI_2_Br films formed stripe structures [38]. Pre-annealing treatment reduced this effect, leading to larger grains, a better crystal structure, improved light absorption, and a longer charge carrier lifetime. Consequently, the PV device’s PCE increased from 12.52% to 13.99%. To study the effect of real-time annealing on CsPbI_2_Br films, films were prepared by thermal co-evaporation, and in situ spectroscopic ellipsometry measurements were conducted [39]. The γ-CsPbI_2_Br phase transitioned to the β phase at 130 °C and to the α phase at 190 °C. At 225 °C, the Cs_4_PbI_4_Br_2_ phase formed. As the temperature increased, the refractive index decreased, the absorption edge slope slowed, and the excitation peak energy shifted. During cooling, the thermo-optic coefficient maintained a linear relationship, while the Urbach energy gap increased linearly. Table 1 summarizes CsPbI_2_Br film deposition techniques and the PV performance of the corresponding PSCs.

High-temperature annealing remains indispensable for achieving phase-pure α-CsPbI_2_Br films with large grains and minimized defects. While these methods ensure excellent thermal stability and reproducibility under controlled lab conditions, their reliance on temperatures > 250 °C limits compatibility with flexible substrates and industrial-scale roll-to-roll processes. Furthermore, iodine volatilization during prolonged annealing can lead to stoichiometric imbalances, necessitating precise environmental controls. Future efforts should explore hybrid approaches combining short high-temperature pulses with post-treatment passivation to mitigate these trade-offs.

### 2.2. Low-Temperature Processing

High-efficiency and stable CsPbI_2_Br PSCs often require a relatively high temperature. However, this condition is incompatible with the fabrication of tandem and flexible PSCs, which are sensitive to high temperatures. Therefore, there is an urgent need for methods that can reduce the temperature threshold for the formation of a cubic-phase perovskite while maintaining efficiency [55,56].

Dimethyl sulfoxide (DMSO) was employed as a solvent to facilitate the formation of CsPbI_2_Br perovskite at low temperatures, which effectively controlled the crystallization kinetics, promoting crystal growth and forming a uniform, dense film (Figure 2c) [40]. Post-annealing at 120 °C optimized the film’s properties, balancing grain growth and surface roughness. This low-temperature process produced flexible CsPbI_2_Br films with high bending stability and attained a PCE of 7.3%. Utilizing DMSO adducts enabled the annealing of CsPbI_2_Br precursor films at low temperatures, resulting in high-quality films with enhanced crystallinity and stability. Subsequently, the DMSO adduct was utilized to fabricate large-area inverted PSCs through the blade coating deposition method [41]. By fine-tuning the processing conditions, setting the blade coating temperature to 80 °C effectively mitigated moisture ingress and Benard–Marangoni instability during the ink-drying process, thereby yielding high-quality films (Figure 2d). As a result, this methodology led to a notable enhancement in the performance of CsPbI_2_Br devices, achieving a PCE of 14.7% for small-area (0.03 cm^2^) devices and 12.5% for large-area (1.0 cm^2^) devices. Similarly, utilizing DMSO as the mediator, a DMSO-mediated one-step solution method was developed to achieve high-quality CsPbI_2_Br films at low temperatures [50]. The XRD analysis revealed that following low-temperature annealing, characteristic black α-phase crystals were formed, suggesting that Pb(SCN)_2_ could facilitate the formation of CsPbI_2_Br films with high crystallinity and smooth and uniform surface morphology.

On the other hand, the iodine-rich precursor HPbI_3+x_ (where x ranges from 0.1 to 0.2) was employed to synthesize a novel precursor in combination with 2CsI and PbBr_2_ [47]. As a result, the perovskite film exhibited thermal stability exceeding one week upon annealing at 100 °C. The CsPbI_2_Br PSCs prepared through this method attained a PCE of 10.56%. ABA, a non-volatile additive, was added to the perovskite precursor solution, resulting in high-quality α-phase perovskite after annealing at 100 °C [42]. This additive slowed the precursor reaction, improving film crystallinity and phase stability and preventing degradation in ambient conditions (Figure 2e). Consequently, the PCE of CsPbI_2_Br PSCs increased to 8.44%. Levulinic acid (LA) was incorporated into the CsPbI_2_Br precursor solution, resulting in the formation of high-quality cubic-phase CsPbI_2_Br at an annealing temperature of 80 °C (Figure 2g) [44]. By utilizing *N*-methyl-2-pyrrolidone (NMP) as the precursor solvent and employing the vacuum-assisted deposition method, a smooth and uniform CsPbI_2_Br film with high crystallinity was successfully fabricated at room temperature [48]. Compared to films annealed at elevated temperatures (280 °C), the room-temperature-annealed film demonstrated superior humidity stability. The PCE of the CsPbI_2_Br PSC device reached 8.67%. Furthermore, polyvinylpyrrolidone (PVP) was employed in a one-step spin-coating procedure to synthesize stable orthorhombic-phase CsPbI_2_Br films under low-temperature conditions [54]. The introduction of PVP modified the crystallization and growth dynamics of the films. CsPbI_2_Br PSCs with a PCE of 10.47% were successfully fabricated at 120 °C.

A gradient thermal annealing (GTA) combined with an antisolvent (ATS) treatment approach was introduced [51]. The sequential annealing process precisely controlled the evaporation rate of residual DMSO and the crystallization process. The ATS treatment further enhanced the CsPbI_2_Br film’s quality and reduced the defect density. Consequently, the CsPbI_2_Br PSCs achieved a PCE of 16.07%, with a stabilized efficiency of 15.75%. Simultaneously, the spin-forced (SF) annealing technique was introduced, which effectively reduced the residual DMSO in the film and ensured a more uniform distribution of colloids in the wet film (Figure 2h) [45]. This, in turn, facilitated the uniform nucleation and growth of the CsPbI_2_Br film, thereby enhancing the film’s quality. Ultimately, CsPbI_2_Br PSCs attained a PCE of 17%, with a stable efficiency of ~16%. The hot-casting method was developed to fabricate CsPbI_2_Br films that are smooth, dense, void-free, and cubic at low temperatures [49]. Consequently, the PCE of CsPbI_2_Br PSCs annealed at 120 °C reached 12.5%. Similarly, using the hot-casting deposition method, a CsPbI_2_Br/DMF precursor solution was deposited onto a substrate preheated to 80 °C, followed by spin coating and low-temperature annealing at 100 °C (Figure 2i) [46]. The preheated substrate facilitated nucleation and crystallization, leading to the successful preparation of large-grain α-CsPbI_2_Br films with smooth and dense surface morphologies. As a result, the PCE was significantly enhanced from 9.82% to 16.44%. Furthermore, the CsPbI_2_Br film underwent an initial pre-annealing step at a relatively low temperature (50 °C), followed by conventional annealing at 160 °C for 10 min [53]. To address the energy level mismatch between CsPbI_2_Br and P3HT, a diphenylamine derivative was utilized as a buffer layer, effectively reducing carrier recombination and thereby minimizing the V_oc_ loss. As a result, the PCE of the undoped P3HT-CsPbI_2_Br PSC reached 15.50%. A vacuum-controlled growth (VCG) method effectively controlled perovskite crystallization, producing high-quality films with larger grains and fewer defects at lower temperatures [52]. Adding polyethyleneimine (PEIE) as a buffer layer improved the PCE of CsPbI_2_Br PSCs to 12.32%. By successfully overcoming the negative impact of water erosion in the dry ink and Benard–Marangoni instability, the ordered crystallization of the ideal halide composition change was achieved during the film formation process (Figure 2f) [43]. As a result, CsPbI_2_Br films with high crystallinity, uniformity, and no pinholes were prepared, and their photophysical and transport properties were excellent. By fabricating high-performance solar cells, the PCE of small-area devices (0.03 cm^2^) reached 14.7%, while that of large-area devices (1.0 cm^2^) achieved 12.5%.

The low-temperature processing of CsPbI_2_Br PSCs involves various strategies to reduce the formation temperature of the cubic phase while maintaining high efficiency and stability. Key mechanisms include using DMSO as a solvent to facilitate low-temperature crystallization, resulting in uniform and dense films. DMSO adducts effectively retard rapid reactions during solvent evaporation, providing a low-energy pathway for perovskite film formation. Post-annealing at moderate temperatures optimizes film properties. Additionally, additives enhance crystallinity and phase stability. Techniques such as spin-forced annealing and hot-casting enable the fabrication of smooth, dense, and void-free films at low temperatures. Pre-annealing steps and the use of buffer layers address energy level mismatches and improve the PCE. These approaches contribute to the development of efficient and stable CsPbI_2_Br PSCs processed at low temperatures.

## 3. CsPbI_2_Br Crystallization Control

### 3.1. Solvent Strategy

There are various methods for fabricating CsPbI_2_Br perovskite films, with solution processing being widely favored due to its low cost, simplicity, and capability for large-scale production. The choice of precursor solvent is a key to obtaining high-quality perovskite films. Table 2 summarizes CsPbI_2_Br film solvent strategies and the PV performance of the corresponding PSCs. Firstly, DMSO is widely employed in manufacturing CsPbI_2_Br perovskite films. For example, DMSO was introduced to partially replace the toxic N, N-dimethylformamide (DMF) [57]. It has a higher boiling point and vapor pressure (189 °C and 126 Pa) than DMF (153 °C and 418 Pa). Increasing the proportion of DMSO lowered the vapor pressure of the mixed solvent, controlling the evaporation rate and affecting the nucleation and crystal growth of perovskites. It also enhanced CsBr dissolution, which was vital for film thickness control. The mechanism by which DMSO induced the room-temperature formation of cubic CsPbI_2_Br was investigated [58]. DMSO molecules spontaneously coordinated with the layered [PbI_6_]^4−^ array in PbI_2_ (or PbBr_2_), forming a CsI-PbI_2_-DMSO intermediate phase. Subsequently, DMSO was gradually displaced due to the stronger affinity of CsI for PbI_2_. This process not only enhanced the solubility of the CsPbI_2_Br precursor but also facilitated the room-temperature formation of cubic CsPbI_2_Br. In another study, a method was described that involved evaporating DMSO before high-temperature annealing to adjust the concentration of the solvent [59]. After vacuum treatment, DMSO partially evaporated, and its low concentration promoted a dense buried interface, preventing small grains and voids at the perovskite/ETL interface. The PCE of CsPbI_2_Br carbon-based PSCs attained 13.46%.

**Figure 3:**
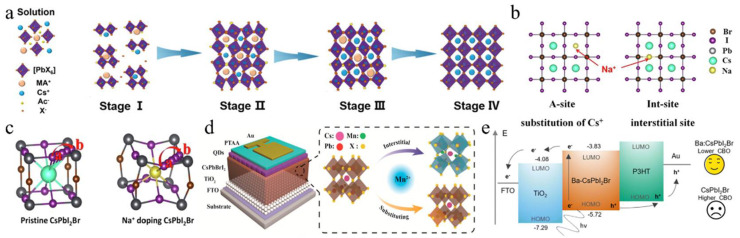
(**a**) CsPbI_2_Br film crystallization kinetics. Reproduced with permission [60]. Copyright 2020, Wiley-VCH. (**b**) Schematic illustration of A-site substitution and the Int-site for Na-doping in CsPbI_2_Br. (**c**) Illustration of the I-diffusion pathway of a → b and the I-diffusion barriers. (**b**,**c**) Reproduced with permission [64]. Copyright 2022, Elsevier. (**d**) Schematic structure of the device and illustration of the Mn^2+^-doping modes: interstitial and substituting. Reproduced with permission [65]. Copyright 2018, American Chemical Society. (**e**) Schematic of the carrier transport mechanism across both interfaces. Reproduced with permission [66]. Copyright 2023, Royal of Society Chemistry.

In addition to DMSO, a variety of alternative solvents can be employed to enhance the quality of perovskite films. For example, the ionic liquid solvent methylaminoacetic acid (MAAc) has been incorporated to modulate the interactions within the CsPbI_3−x_Br_x_ perovskite structure (Figure 3a) [60]. The strong interaction between C=O and lead (Pb^2+^), along with the formation of N-H…I hydrogen bonds, stabilized the perovskite precursor solution and inhibited crystallization. This method provided CsPbI_2_Br PV devices with an efficiency of up to 15.82%. Apart from this, a low-toxicity and high-volatility solvent, acetone, was introduced into the precursor [61]. Adding acetone improved the wetting of the perovskite precursor on TiO_2_ and enhanced the interaction between PbI_2_ and DMSO, lowering the crystallization activation energy and enabling the rapid formation of α-phase CsPbI_2_Br at 40 °C. Therefore, the device achieved not only an excellent PCE of 16.03% but also enabled the efficient low-temperature preparation of α-phase CsPbI_2_Br.

The high boiling point and low evaporation rate of commonly used solvents delay the crystallization of perovskite films, which makes it difficult to obtain uniform and pinhole-free perovskite films. Therefore, low-boiling-point antisolvent engineering is introduced for research. The environmentally friendly solvent, ethyl acetate (EA), has been successfully developed as an effective antisolvent [62]. Compared to films that use chlorobenzene (CB) as the antisolvent, CsPbI_2_Br films with EA demonstrated superior crystallinity, larger grain sizes, and a denser, more uniform surface morphology. CsPbI_2_Br PSCs with the EA antisolvent achieved a PCE of 10.0% and exhibited outstanding long-term stability. In addition, methoxyacetone (MeOAc) served as an antisolvent to enhance perovskite nucleation and crystallization [63]. Finally, the CsPbI_2_Br PSCs attained a high PCE of 15.86%, an enhanced V_oc_ of 1.23 V, and a high FF of 0.82.

The mechanism of the solvent strategy in fabricating high-quality CsPbI_2_Br perovskite films primarily involves the careful selection and manipulation of precursor solvents. DMSO is widely used to control the evaporation rate of the solvent mixture, influencing nucleation and crystal growth. Alternative solvents such as MAAc and acetone are also employed to modulate interactions within the perovskite structure and enhance precursor solubility. Low-boiling-point antisolvents can also enhance perovskite nucleation and crystallization, resulting in films with superior crystallinity, larger grain sizes, and more uniform and denser surface morphologies. The application of these solvents and antisolvents helps to significantly enhance the PCE and stability of CsPbI_2_Br PSCs.

**Table 2:** CsPbI_2_Br film solvent strategies and the PV performance of the corresponding PSCs.

Device Architecture	Active Area (cm^2^)	PCE (%)	J_sc_ (mA/cm^2^)	V_oc_ (V)	FF (%)	Stability	Year	Refs.
FTO/c-TiO_2_/mp-TiO_2_/CPI_2_/Spiro-OMeTAD/Ag	0.12	12.52	13.56	1.24	74.3	No detectable degradation for more than 500 h	2018	[57]
PET/ITO/NiO_x_/CsPbI_2_Br/C_60_/BCP/Ag	0.118	7.3	11.5	0.97	0.65	/	2018	[58]
FTO/c-TiO_2_/CsPbI_2_Br/carbon	0.12	10.0	13.54	1.15	64.2	Retained 94% of its initial PCE after being exposed to air with 15–30% RH for 39 d	2018	[62]
FTO/SnO_2_/CsPbI_2_Br/Spiro-OMeTAD/MoO_3_/Ag	0.05	15.83	16.52	1.32	72.40	Maintained 85% of its initial efficiency after being exposed to a N_2_ atmosphere for 1500 h	2020	[60]
FTO/c-TiO_2_/CsPbI_2_Br/Spiro-OMeTAD/Au	0.09	16.03	15.98	1.270	79.00	Retained more than 90% of its initial efficiency after 500 h of thermal aging at 85 °C in a N_2_-filled glove box	2020	[61]
ITO/SnO_2_/TiO_2_/CsPbI_2_Br/Spiro-OMeTAD/MoO_3_/Ag	0.075	15.86	15.67	1.23	82.29	Kept ~95% of its initial PCE after 1 m storage in a N_2_-filled glove box without any encapsulation	2020	[63]
FTO/TiO_2_/CsPbI_2_Br/PCBM/carbon	0.9	13.46	14.71	1.19	76.28	Almost no decay after 33 d of storage at 25 °C with an RH of 25%	2023	[59]

### 3.2. Component Engineering

#### 3.2.1. A-Site

Perovskite films are the essential component of PSCs, and optimizing these films is a key focus in the study of PSCs. The incorporation of alkali metal cations into the A-site of the inorganic perovskite lattice has been extensively studied. Table 3 summarizes the A/B/X-site-doping engineering of CsPbI_2_Br films and the PV performance of the corresponding PSCs. This section mainly discusses potassium ions (K^+^), rubidium ions (Rb^+^), sodium ions (Na^+^), and FA^+^. Nam et al. were the first to report the incorporation of K cations into CsPbI_2_Br perovskite, leading to the formation of Cs_1−x_K_x_PbI_2_Br [67]. The introduction of K^+^ cations resulted in a reduction in the volume of the PbX_6_ octahedra, thereby enhancing phase stability. Consequently, a device utilizing Cs_0.925_K_0.075_PbI_2_Br achieved a PCE of 10.0%. Later, the thermal air method and Rb^+^, introduced as A-site dopants, were simultaneously applied, enhancing phase stability and reducing defect density [68]. As a result, the Cs_0.99_Rb_0.01_PbI_2_Br PSCs reached a PCE of 17.16% and exhibited good thermal stability when combined with poly(3-hexylthiophene-2,5-diyl) (P3HT). At the same time, Zhang et al. used Rb^+^ doping in Cs_1−x_Rb_x_PbI_2_Br perovskite and employed amino bromide (GABr) post-treatment [69]. The addition of smaller Rb cations enhanced the structural stability of the perovskite by promoting crystal contraction. Modification of GABr led to a 2D/3D heterostructure with enhanced crystallinity, improved surface morphology, and a reduced trap-state density. Consequently, the Cs_0.9_Rb_0.1_PbI_2_Br PSC acquired a PCE of 15.6%. Sodium thiocyanate (NaSCN) was introduced as a synergistic passivator for both metal and halide ions (Figure 3b,c) [64]. The incorporation of Na^+^ at the A-site of CsPbI_2_Br increased the iodide diffusion barrier energy from 0.229 eV to 0.401 eV, effectively suppressing iodide-related defects. The SCN^−^ anion played a pivotal role in modulating the crystal growth dynamics, resulting in enhanced crystallinity and larger grain sizes in the CsPbI_2_Br films. Consequently, the Cs_0.995_Na_0.005_PbI_2_Br PSCs achieved a PCE of 14.19%. Recently, phase-pure Cs-rich FA-Cs perovskite Cs_1−x_FA_x_PbI_2_Br (0 ≤ x ≤ 0.6) films were first synthesized through the use of a PbI_2_ (DMSO) and PbBr_2_ (DMSO) mixture [70]. As the concentration of FA^+^ increased, the grain size of the perovskite also expanded. When the FA^+^ content surpassed 30%, the reduced nucleation Gibbs free energy barrier facilitated agglomeration, leading to decreased film coverage and the formation of multiple voids. The Cs_0.7_FA_0.3_PbI_2_Br devices achieved an efficiency of 14.55%. The incorporation of alkali metal cations into the A-site of inorganic perovskite lattices in PSCs is an effective strategy to enhance phase stability, reduce defect density, and improve crystallinity, ultimately leading to improved device performance and efficiency.

A-site doping with alkali metals (e.g., K^+^, Rb^+^) effectively stabilizes the perovskite lattice and reduces defect densities. However, excessive doping (>5 mol%) introduces strain and phase segregation, undermining long-term stability. For instance, FA^+^ incorporation beyond 30% caused void formation due to reduced nucleation barriers. A key strength of this strategy lies in its simplicity and compatibility with existing fabrication workflows, but precise doping thresholds must be empirically determined for each cation to balance performance and stability.

#### 3.2.2. B-Site

In addition to A-site doping, B-site doping also significantly affects the crystal growth and film quality of CsPbI_2_Br perovskite [84,85,86,87]. Up to now, several divalent metal cations have been utilized for B-site doping, including strontium (Sr^2+^), germanium (Ge^2+^), manganese (Mn^2+^), zinc (Zn^2+^), barium (Ba^2+^), and iron (Fe^2+^). For instance, Hayase’s group first reported the preparation of CsPb_1−x_Ge_x_I_2_Br perovskite materials by substituting different amounts of Ge^2+^ for Pb^2+^ [88]. As the Ge content increased, the valence band and conduction band shifted upwards, thereby improving the carrier mobility and significantly enhancing the V_oc_ and FF. Meanwhile, Mn^2+^ was also doped to increase the lattice constant and control the growth of the film. During the growth process, Mn^2+^ occupied the vacancies in the CsPbI_2_Br lattice, effectively inhibiting nucleation and slowing down the growth rate (Figure 3d) [65]. Moreover, excess Mn^2+^ accumulated at the grain boundaries, resulting in an effective passivation effect. As a result, the CsPb_0.98_Mn_0.02_I_2_Br PSCs showed a V_oc_ of 1.172 V and a J_sc_ of 14.37 mA/cm^2^. Stable and abundant Zn^2+^ was used to reduce the Pb^2+^ content in CsPbI_2_Br perovskite [72]. Zn’s higher chemical activity compared to Pb led to stronger coordination with Br/I, which slowed crystal growth and resulted in larger grains with better orientation. Cs-Zn-I/Br compounds helped to passivate grain boundaries, which reduced trap states and enhanced charge transport. Consequently, this increased the PCE of CsPb_0.9_Zn_0.1_I_2_Br PV cells to 13.6%, significantly higher than the 11.8% efficiency of pure CsPbI_2_Br PSCs. FeCl_2_ was introduced into the CsPbI_2_Br precursor to stabilize the α-CsPbI_2_Br phase and prevent the formation of non-perovskites caused by the reduction of grain size due to Fe^2+^ [78]. The addition of FeCl_2_ effectively adjusted the energy levels, improved the built-in potential (V_bi_), and decreased defect states in the perovskite, resulting in a record-breaking PCE of 17.1% and a V_oc_ of 1.31 V in the CsPb_0.995_Fe_0.05_I_2_Br device.

In addition to transition metals, the alkaline earth metals strontium (Sr^2+^) and barium (Ba^2+^) can also be used as B-site dopants. For example, incorporating a low concentration of the less toxic strontium (Sr) to partially replace Pb in CsPb_1−x_Sr_x_I_2_Br was first demonstrated by low-temperature processing, in which strontium concentrated on the surface of the perovskite film, acting as a passivation agent [71]. The optimal CsPb_0.98_Sr_0.02_I_2_Br PSC achieved a PCE of 11.3%. A hot air treatment method was utilized to partially substitute Pb^2+^ with Sr^2+^, resulting in lattice contraction [79]. This process not only improved the surface morphology and enhanced the stability of the photoactive black phase but also mitigated interface charge accumulation losses by elevating the conduction band minimum (CBM) energy level. As a result, the CsPb_0.98_Sr_0.02_I_2_Br PSC attained a PCE of 16.61%. The impact of incorporating Ba^2+^ into CsPbI_2_Br was investigated [73]. It was found that barium did not integrate into the perovskite lattice but instead induced phase segregation, leading to a modified iodine (I) to bromine (Br) ratio relative to the original precursor stoichiometry. This resulted in a decrease in the bandgap of the perovskite phase, effectively inhibiting non-radiative recombination. Ultimately, the CsPb_0.8_Ba_0.2_I_2_Br PSCs exhibited a PCE of 14.0% and a high V_oc_ of 1.33 V. Furthermore, a low concentration of Ba^2+^ was also doped into CsPbI_2_Br, yielding a mixed CsPb_1_−_x_Ba_x_I_2_Br perovskite film (Figure 3e) [66]. The CsPb_0.95_Ba_0.05_I_2_Br/TiO_2_ interface showed a type II staggered band alignment with a conduction band offset (CBO) of 0.25 eV, lower than the 0.48 eV CBO at the CsPbI_2_Br/TiO_2_ interface, which resulted in a lower energy barrier for electron transfer from the Ba-CsPbI_2_Br layer to the TiO_2_ layer, thereby enhancing charge transport efficiency.

**Figure 4:**
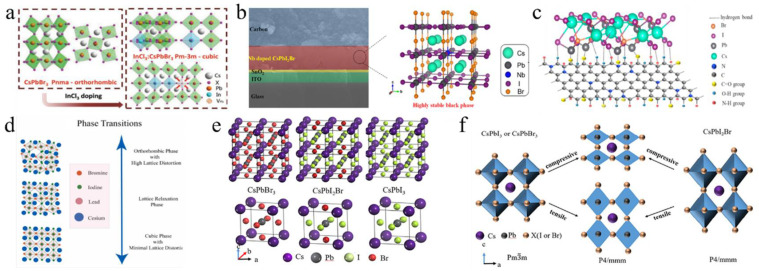
(**a**) Evolution of the crystal structure and space group of a CsPbBr_3_ single crystal with an InCl_3_ dopant. Reproduced with permission [26]. Copyright 2018, Wiley-VCH. (**b**) Cross-sectional SEM image of the carbon electrode-based CsPbI_2_Br perovskite device and the schematic illustration of Nb^5+^ replacing Pb^2+^. Reproduced with permission [76]. Copyright 2019, American Chemical Society. (**c**) Schematic illustration of the interaction between N-GQDs and CsPbI_2_Br film. Reproduced with permission [80]. Copyright 2023, Elsevier. (**d**) The lattice relaxation for the various phases observed with temperature increments. Reproduced with permission [90]. Copyright 2024, Elsevier. (**e**) The crystal structure of CsPbI_3−x_Br_x_. (**f**) Schematic illustration of the generation of tensile and compressive strains within a perovskite film. (**e**,**f**) Reproduced with permission [91]. Copyright 2024, American Institute of Physics.

Some metal cations with high valency (>2) are used as additives, which tends to alter the concentration and type of the majority charge carriers in the perovskite, thereby significantly influencing the optoelectronic properties of CsPbI_2_Br films. For example, the perovskite parent lattice was doped with InCl_3_ through a novel thermal radiation annealing method, employing B-site and X-site co-doping engineering (Figure 4a) [89]. The co-doping of In^3+^ and Cl^−^ caused a phase transition from orthorhombic (Pnma) to cubic (Pm-3m), thereby increasing spatial symmetry and enabling the formation of a pure and stable α phase of CsPbI_2_Br perovskite. The InCl_3_:CsPbI_2_Br PSC resulted in a PCE of 13.74% for the small-area device (0.09 cm^2^), while the large-area device (1.00 cm^2^) achieved a PCE of 11.4%. Subsequently, calcium chloride (CaCl_2_) and indium chloride (InCl_3_) were added to the precursor solution to produce high-quality double-doped CsPbI_2_Br films [82]. The CaCl_2_ additive helped to isolate moisture by forming hydrates at the surfaces and grain boundaries of the perovskite. Meanwhile, InCl_3_ improved the optoelectronic properties by partially substituting Pb^2+^ with In^3+^. This dual doping greatly reduced phase separation by diminishing electron–phonon coupling and increasing the activation energy for ionic migration. The dual-doped Ca- and In-CsPbI_2_Br PSC achieved a PCE of 15.51%. In a recent study, Eu(Ac)_3_ was introduced into CsPbI_2_Br perovskite to create high-quality perovskite films with low defect densities and prolonged carrier lifetimes [74]. The smaller Eu^3+^ and Ac^−^ ions substituted for Pb^2+^ and I^−^, respectively, which helped stabilize the α phase of CsPbI_2_Br perovskite films. The Eu(Ac)_3_:CsPbI_2_Br solar cells achieved a high efficiency of 15.25%. Simultaneously, Eu-doped CsPbI_2_Br perovskite could stabilize its α phase at room temperature [75]. The incorporation of europium altered the bandgap structure, significantly decreasing non-radiative recombination. The PSC with CsPb_0.95_Eu_0.05_I_2_Br yielded a PCE of 13.71% and maintained a stable power output of 13.34%.

Additionally, doping the B-site with tetravalent and pentavalent cations, such as zirconium ions (Zr^4+^) and niobium ions (Nb^5+^), has been utilized to enhance the properties of CsPbI_2_Br perovskite. Zirconium tetrachloride (ZrCl_4_) was incorporated into the CsPbI_2_Br film, enabling Zr^4+^ to partially substitute Pb^2+^ at the B-site [83]. Due to the smaller ionic radius of Zr^4+^ compared to Pb^2+^, the tolerance factor t of CsPb_0.996_Zr_0.004_I_2_Br increased from 0.85 to 0.95. Furthermore, the interaction between Zr^4+^ and CsPbI_2_Br led to the contraction of [PbX_6_]^4−^, which improved phase stability, suppressed the transition from the α phase to the δ phase, and enhanced both humidity and thermal stability. The incorporation of Nb^5+^ ions into the CsPbI_2_Br perovskite structure effectively stabilized the optically active α-CsPbI_2_Br phase (Figure 4b) [76]. Moreover, the positive charge introduced by Nb^5+^ could be balanced by the formation of Pb^2+^ vacancies and the presence of excess halide anions at the perovskite sites. This resulted in reduced charge recombination in CsPb_0.995_Nb_0.005_I_2_Br PSCs, achieving a PCE of 10.42% with minimal hysteresis. In a subsequent study, Nb^5+^-doped PSCs were fabricated using a thermal air method, which reduced charge recombination and effectively eliminated hysteresis in the cells [77]. The addition of NbCl_5_ changed the chemical state of the [PbX_6_]^4−^ octahedra, enhanced the interactions between Pb-I and Cs-I, and increased the accumulation of Cl^−^ ions at the surface, thereby improving the hydrophobic properties of CsPbI_2_Br films. Consequently, the CsPb_0.995_Nb_0.005_I_2_Br PSC reached a PCE of 10.42%.

B-site doping with various metal cations is a crucial strategy for optimizing the performance and stability of CsPbI_2_Br PSCs. The incorporation of B-site dopants in CsPbI_2_Br perovskite substantially affects crystal growth and film morphology.

#### 3.2.3. X-Site

The integration of various additives and doping strategies has shown significant potential in enhancing the performance and stability of CsPbI_2_Br PSCs. The effects of binary additives, namely NaCl and nitrogen-doped graphene quantum dots (N-GQDs), were investigated to improve the photovoltaic performance of CsPbI_2_Br_x_Cl_1−x_ perovskite films (Figure 4c) [80]. The introduction of Cl^−^ ions aligned the energy levels of CsPbI_2_Br, thereby facilitating hole transport and extraction. Meanwhile, N-GQDs acted as an electronic bridge, promoting electron conduction and preventing electron annihilation at grain boundaries. These modifications resulted in a substantial increase in the V_oc_ to 1.24 V and achieved a PCE of 15.37%. The incorporation of lead chloride (PbCl_2_) into CsPbI_2_Br not only improved grain orientation and slowed down nucleation but also resulted in larger grain sizes [92]. Additionally, the introduction of Cl^−^ altered the emission wavelength and reduced the lattice parameter, thereby enhancing film stability and leading to superior PV performance.

Further optimization of the perovskite structure was achieved through the addition of excess CsBr into the precursor solution and the use of an ultrathin MgF_2_ buffer layer at the ETL/Ag interface [81]. The excess CsBr improved crystallinity and reduced defect states, while the MgF_2_ layer formed an ohmic contact, thereby eliminating charge accumulation at the interface. These modifications resulted in a PCE of 15.6% and provided excellent thermal and moisture stability for CsPbI_2_Br PSCs. Khalid Javed’s group concentrated on modifying the molar ratios of CsBr to PbI_2_ in the precursor solution, which identified deep-level charge traps that contributed to the degradation of the perovskite material (Figure 4d) [90]. Finally, a 1.05:1 ratio of CsPbI_2_Br in the precursor resulted in superior stability and an improved PCE under ambient air conditions. First-principles calculations investigated how strain affects the phase stability and optoelectronic properties of CsPbI_3−x_Br_x_ (Figure 4e,f) [91]. Compressive stress reduced the bandgap and enhanced light absorption, improving performance. Tensile stress caused a cubic-to-tetragonal phase transition, reducing light absorption. The halide mixture in CsPbI_3_−_x_Br_x_ stabilized the tetragonal phase, indicating that controlling stress during fabrication could optimize PV performance and phase stability in PSCs. These studies emphasize the crucial role of additive engineering, doping techniques, and stress management in the development of high-performance, stable PSCs.

### 3.3. Additive Engineering

To improve the crystallization quality of perovskite films, various additives are incorporated into the perovskite precursor solution [93,94]. Table 4 summarizes CsPbI_2_Br dopant strategies and the PV performance of the corresponding PSCs.

**Liquid-type additives:** First of all, various liquid-type additives have been introduced into several studies. For example, small carbon chain molecules (diiodomethane (DIM), dibromoethane (DBM), and dichloromethane (DCM)) were employed as liquid additives in the perovskite precursor solution [98]. Among these, DIM exhibited superior performance by effectively passivating uncoordinated Pb^2+^ ions, promoting oriented crystal growth, suppressing halide ion vacancies, and mitigating surface defects. Consequently, this approach resulted in a CsPbI_2_Br PSC achieving a PCE of 16.42%. In a similar approach, butyl-3-methylimidazolium tetrafluoroborate (BMIMBF_4_), an ionic liquid, was utilized as an additive in the perovskite precursor solution [99]. The BMIM^+^ and BF_4_^−^ ions coordinated with uncoordinated Pb^2+^ and I^−^ ions, helping to reduce defect density and allowing excitons to predominantly exist as free carriers. Additionally, these ions stabilized the structure by preventing the tilting of the [PbI_6_]^4−^ octahedra and enhanced moisture resistance through the incorporation of superhydrophobic fluorine groups, ultimately improving the film’s performance.

**Figure 5:**
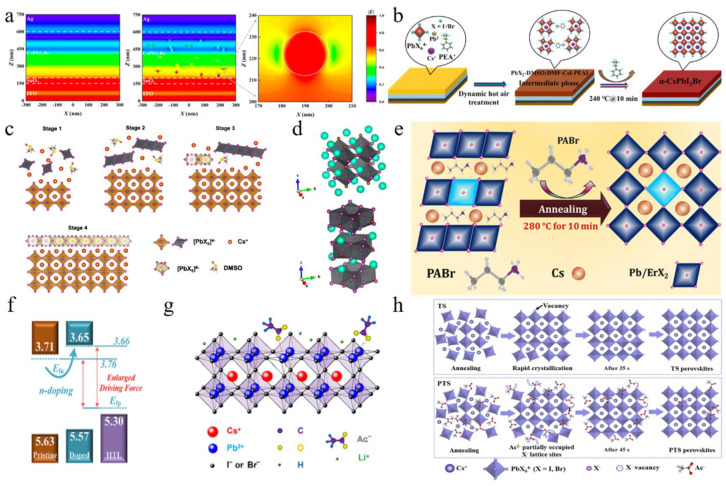
(**a**) Distribution of electric field strength in the x-z cross-section of the device without and with the addition of dispersed SiO_2_ nanoparticles at the incident light wavelength of 550 nm, respectively. Reproduced with permission [101]. Copyright 2023, American Chemical Society. (**b**) Schematic representation of a possible mechanism. Reproduced with permission [102]. Copyright 2023, Royal of Society Chemistry. (**c**) Schematic diagram of the formation process of the target BHJ film. (**d**) Crystal structures of CsPbI_2_Br and CsPb_2_I_4_Br. (**c**,**d**) Reproduced with permission [104]. Copyright 2023, American Chemical Society. (**e**) Growth mechanism and chemical composition. Reproduced with permission [105]. Copyright 2023, Royal of Society Chemistry. (**f**) Energy level diagrams for pristine and LiAc-doped CsPbI_2_Br perovskites and Spiro-TTB HTL. (**g**) Schematic of the doping mechanism of LiAc into CsPbI_2_Br perovskite. (**f**,**g**) Reproduced with permission [96]. Copyright 2023, Elsevier. (**h**) Schematic diagram of the TS and PTS perovskite crystallization processes during the thermal annealing process. Reproduced with permission [127]. Copyright 2024, Elsevier.

**Nanometer-sized additives:** Some studies have introduced nanometer-sized additives to enhance PV performance. CsPbBr_3_ nanocrystals (NCs) were used to improve electron transport within the perovskite layer by leveraging Br–I interactions and lattice contractions [100]. Similarly, SiO_2_ nanoparticles were incorporated into the active layer of CsPbI_2_Br PSCs, which not only improved the electric field and increased light absorption through Rayleigh scattering but also enhanced crystallinity, reduced trap density, and improved resistance to moisture and silver diffusion (Figure 5a) [101]. Consequently, these modifications resulted in a PCE of 15.32% and a J_sc_ of 16.21 mA/cm^2^. In a subsequent study, two-dimensional tin selenide (SnSe) nanosheets were incorporated into the CsPbI_2_Br precursor via ultrasonic exfoliation [114]. This approach not only mitigated defects arising from disordered crystallization but also served as a crystallization template, thereby enhancing the orientation of the α-CsPbI_2_Br (200) plane. The resultant improvements in crystallinity and energy level alignment led to enhanced n-type characteristics, achieving a PCE of 14.24% and a V_oc_ of 1.22 V. These studies demonstrate the significant role of various additives in enhancing the efficiency and stability of CsPbI_2_Br PSCs. These additives could enhance electron transport within the perovskite layer, improve crystallinity by serving as crystallization templates, and facilitate interface engineering to optimize the transfer of charge carriers and reduce energy loss.

**Halide-based additives:** Phenylethylamine iodide (PEAI) was incorporated into perovskite precursors as an additive to modulate the crystallization process (Figure 5b) [102]. The introduction of PEAI facilitated the formation of a PbX_2_-DMSO:DMF-PEAI-CsI intermediate phase, which effectively decelerated crystal growth. This phenomenon promoted Ostwald ripening, resulting in the formation of larger grains with reduced voids. Using this method, a CsPbI_2_Br device achieved a PCE of 17.40%. Guanidinium iodide (GAI), as a volatile additive, and phenyltrimethylammonium chloride (PTACl), as a passivation agent, were developed to effectively increase the size of perovskite crystals [103]. GAI interacted with Cs^+^ and GA^+^ ions through a cation-exchange process, which delayed the reaction between cations and anions. During the annealing process, GAI was completely sublimed. Simultaneously, chloride ions from PTACl were incorporated into the CsPbI_2_Br lattice, creating a hydrophobic surface that effectively reduced moisture-related degradation. This optimization resulted in a V_oc_ of 1.34 V and a PCE of 16.88% for CsPbI_2_Br devices. A 2D CsPb_2_I_4_Br layer was successfully constructed by adding excess PbI_2_, which was integrated into the 3D CsPbI_2_Br perovskite framework, forming a fully inorganic 2D/3D CsPb_2_I_4_Br/CsPbI_2_Br bulk heterojunction (BHJ) (Figure 5c,d) [104]. In this structure, Pb^2+^ ions were placed at the center of the [Pb_2_X_5_]^−^ layer, whereas X^−^ ions mainly constituted the surface. This chloride-rich surface effectively passivated iodine vacancy defects in CsPbI_2_Br, significantly reducing non-radiative recombination losses. As a result, the V_oc_ and PCE of the PSCs were enhanced to 1.32 V and 15.25%, respectively. Erbium-doped (ErCl_3_) CsPbI_2_Br perovskites were synthesized with propylammonium bromide (PABr) as an additive (Figure 5e) [105]. The incorporation of PABr effectively retarded the crystallization process, thereby promoting the formation of micron-sized crystals. Moreover, Er^3+^ ions partially substituted Pb^2+^ sites, leading to lattice contraction within the perovskite structure. The addition of PABr significantly enhanced surface morphology, improved crystallinity, and reduced the defect density in the films. As a result, the CsPbI_2_Br device exhibited superior long-term stability and achieved a PCE of 16.74%. These additives played crucial roles in regulating crystal growth, promoting the formation of larger grains with reduced voids, and enhancing crystallinity.

**Salt-based additives:** Salt-based additives are an effective way of optimizing the crystallization of CsPbI_2_Br perovskite films. Lithium acetate (LiAc) was incorporated into the precursor to inhibit the formation of the intermediate product CsBr (Figure 5f,g) [96]. By doping with LiAc, CsAc was formed from CsBr, which helped to suppress the formation of CsBr, slowed down crystallization, minimized phase separation, and ultimately improved film quality. Ac^−^ ions coordinated with Pb^2+^, while Li^+^ accumulated on the surface of the perovskite. This interaction raised the Fermi level of CsPbI_2_Br to the edge of the valence band, reducing trap-assisted recombination losses and enhancing charge extraction. As a result, this approach achieved a V_oc_ of 1.30 V and a PCE of 16.05%. During the annealing process, CsPbI_2_Br underwent a phase transition from α → δ → α. The introduction of formamidine acetate (FAAc) effectively suppressed the transition of the intermediate phase [97]. Consequently, the photovoltaic performance of CsPbI_2_Br achieved a J_sc_ of 16.81 mA/cm^2^, a V_oc_ of 1.24 V, an FF of 0.78, and a PCE of 16.36%. Potassium trifluoroacetate (K-TFA) was employed to modify δ-phase CsPbI_2_Br films [106]. The carboxyl groups in K-TFA bound to uncoordinated Pb^2+^ ions, while the incorporation of K^+^ ions promoted heterogeneous nucleation during the δ → α phase transition. This interaction accelerated nucleation and reduced the energy barrier for the phase transformation. Additionally, K-TFA not only passivated surface defects but also facilitated secondary crystal growth during the phase transition. Potassium acetate (KAc) functioned as both a cathode buffer layer (CBL) and an additive in the fabrication of PSCs [107]. When utilized as an additive, K^+^ ions occupied interstitial sites within the cubic lattice, leading to lattice expansion. Meanwhile, Ac^−^ ions passivated uncoordinated Pb^2+^ and Sn^4+^ ions, thereby elevating the CBM and Fermi level, which effectively reduced the interfacial energy barrier. The synergistic effects of these modifications not only enhanced the film quality but also increased the PCE to 15.71%. Additionally, the device demonstrated excellent thermal stability. Analogously, the incorporation of lead acetate (PbAc_2_) via a twice spin-coating (PTS) process was employed to fabricate the perovskite films (Figure 5h) [127]. The Ac^−^ ligands could retard the crystallization kinetics of the perovskite, resulting in thicker and denser CsPbI_2_Br films. Moreover, the lone pair electrons from the oxygen atoms in the Ac^−^ groups could coordinate with undercoordinated Pb^2+^ ions, effectively filling halide vacancies and thereby reducing defect density. Consequently, the CsPbI_2_Br devices achieved a PCE of 16.19%. Afterward, by incorporating methylamine acetate (MAAc) into the perovskite precursor solution, large-scale fabrication of the film became feasible [116]. The N-H…H interactions facilitated rapid crystallization through strong ionic bonding, leading to the formation of the intermediate phase MA_x_Cs_1_−_x_PbI_2_Br. High-temperature annealing subsequently resulted in the pure phase CsPbI_2_Br, thereby preventing its direct and rapid formation. The fabricated PSCs achieved record-high efficiencies of 18.14% for an active area of 0.1 cm^2^ and 16.46% for an active area of 1 cm^2^.

**Figure 6:**
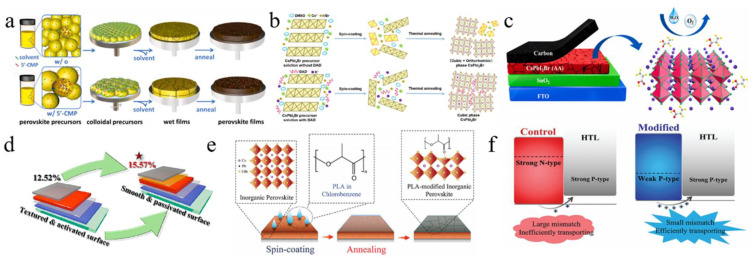
(**a**) Schematic depiction of the formations of perovskite films from colloidal precursors without and with 5′-CMP. Reproduced with permission [109]. 20Copyright 2024, Elsevier. (**b**) Schematic depiction of the formations of perovskite films from CsPbI0_2_Br precursors without and with DAD. Reproduced with permission [110]. Copyright 2023, American Chemical Society. (**c**) Illustration of the interaction between CsPbI_2_Br perovskite and AA. Reproduced with permission [111]. Copyright 2023, Elsevier. (**d**) The PCE of the PSCs rose from 12.52% to 15.57%. Reproduced with permission [38]. Copyright 2023, American Chemical Society. (**e**) Illustration of PLA modification. (**f**) Its effect on energy level alignment. (**e**,**f**) Reproduced with permission [112]. Copyright 2023, Wiley-VCH.

In addition to acetate, other types of salts are utilized as additives. For example, the COOH^−^ ion, functioning as a pseudo-halide anion, interacted with Pb^2+^ to effectively passivate halide anion vacancies [108]. Sodium formate (NaFo) was incorporated into the precursor solution of CsPbI_2_Br, where Na^+^ ions mitigated defects at the SnO_2_/PVK interface. Consequently, the performance of CsPbI_2_Br PSCs was enhanced, resulting in an improved FF of 0.845. Moreover, 5′-cytidine monophosphate (5′-CMP) was introduced as an additive in the inorganic perovskite precursors, which facilitated precursor aggregation, forming larger colloidal clusters that reduced nucleation sites and promoted the growth of larger grains (Figure 6a) [109]. The strong interaction between 5′-CMP and PbI_2_ alleviated residual strain and lowered the perovskite film’s modulus, thereby enhancing mechanical stability. As a result, the optimized CsPbI_2_Br device achieved efficiencies of 15.94% and 33.22% under single-light and white LED (WLED) illumination, respectively. Then, the water-insoluble 2,2′-dihydroxy-4,4′-dimethoxy-5,5′-disulfobenzophenone disodium salt (BP-9) was incorporated into the CsPbI_2_Br precursor solution, where Na^+^ ions and -OH groups helped passivate electron-rich and grain boundary defects in the perovskite film [117]. The negatively charged regions around the carbonyl and sulfonate groups could chelate uncoordinated Pb^2+^ ions, aiding crystallization and defect passivation. The strong chelation between BP-9 and water-soluble Pb^2+^ ions formed insoluble substances, preventing lead diffusion in the environment. The optimized PSC achieved an efficiency of 17.11%. Subsequently, cesium cyclopropanecarboxylic acid (C3) was used as a complexing agent to enhance the moisture resistance of perovskite materials [115]. The introduction of C3 altered the evaporation enthalpy of volatile byproducts derived from DMA acid, thereby shifting water-related reactions towards DMA acid. This modification rendered the target CsPbI_2_Br perovskite less susceptible to ambient humidity. As a result, CsPbI_2_Br PSCs attained efficiencies exceeding 17% under an RH of 45%. Then, diphenylamine-1,8-disulfonic acid potassium salt (DAD) was incorporated into the precursor solution to optimize CsPbI_2_Br PSCs (Figure 6b) [110]. The strong coordination between the SO_3_^2−^ and C=O groups in DAD with Pb^2+^ effectively inhibited the formation of the δ phase and halogen segregation. This raised the valence band maximum (VBM) and brought the Fermi level (E_f_) closer to the CBM, thereby enhancing the V_bi_ and increasing V_oc_. Additionally, K^+^ ions from DAD also contributed to the stability and performance of the α phase. Finally, the best efficiency was 17.38%, with an FF of 0.836.

These salt-based additives play a crucial role in modifying the crystallization process, passivating defects, and enhancing the electronic properties of CsPbI_2_Br perovskite films, ultimately leading to improved PV performance and PSC stability.

**Molecule-based additives:** Molecular-based additives have also garnered considerable attention. The carbonyl (C=O) groups in acrylic acid (AA), the lone electron pairs of various oxygen-containing functional groups, and N atoms in hydrogel-based carbon (HBC) could form coordination bonds with lead ions, significantly reducing the defect state density at the grain boundaries (Figure 6c) [111]. Additionally, the nitrogen element in HBC could provide extra electrochemical active sites, thereby enhancing conductivity. As a result of the AA and HBC modifications, the optimal PCE for CsPbI_2_Br PSCs reached 12.71%. Analogously, oleic acid (OA) was employed to eliminate stripes and modify the surface of stripe-free perovskite films by linking stripe formation to iodine/bromine homogenization during annealing [38]. OA molecules bound to uncoordinated lead ions and their long alkyl chains enhanced the hydrophobicity of the films. As a result, the PCE of the PSCs increased from 12.52% to 15.57%, thereby improving the long-term stability of the devices. Furthermore, multifunctional 2,5-thiophene dicarboxylic acid (2,5-TDCA) was utilized to modify interface defects [118]. The 2,5-TDCA molecule contains C=O and -OH functional groups. Specifically, the oxygen atom in the C=O group coordinated with uncoordinated Pb^2+^ ions, while the -OH group interacted with halide I^−^ ions on the perovskite surface via hydrogen bonding. Consequently, this modification significantly suppressed non-radiative recombination, resulting in a PCE of 13.42% for CsPbI_2_Br PSCs with carbon electrodes.

**Figure 7:**
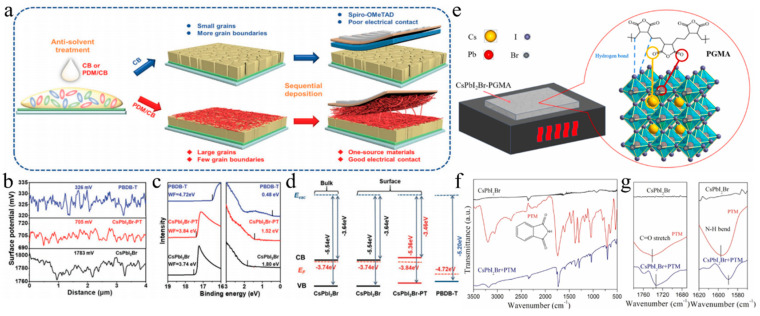
(**a**) Schematic diagram of the one-source strategy for the preparation of perovskite films. (**b**) Surface potential line profiles. (**c**) UPS cutoff edge and valence band spectra. (**d**) Schematic energy level diagrams of a CsPbI_2_Br film, CsPbI_2_Br-PT film, and PBDB-T HTL. (**a**–**d**) Reproduced with permission [95]. Copyright 2021, Wiley-VCH. (**e**) The bonding mechanism diagram of the PGMA additive and the CsPbI_2_Br perovskite. Reproduced with permission [119]. Copyright 2024, Elsevier. (**f**) FTIR plots of the pure CsPbI_2_Br precursor, PTM, and the PTM-CsPbI_2_Br precursor in full scanning range. (**g**) The selected fingerprint range. (**f**,**g**) Reproduced with permission [113]. Copyright 2023, Elsevier.

Not only are organic molecules getting considerable attention, but polymer materials are also being introduced into some research. For example, polylactic acid (PLA) was used to modify perovskite films (Figure 6e,f) [112]. The C=O in PLA strongly interacted with Pb^2+^, passivating surface defects and promoting secondary crystallization post-annealing. This increased the VBM of PVK, shifted the Fermi level toward the valence band, and transformed the PVK surface from n-type to p-type, improving energy level alignment. Additionally, the PLA coating enhanced air stability in CsPbI_2_Br PSCs. PBDB-T served as both a dopant and the HTL in CsPbI_2_Br (Figure 7a–d) [95]. This dual role promoted perovskite growth, increased grain size, reduced defects, and enhanced conductivity. The PDM formed a gradient distribution within the CsPbI_2_Br film and a fingerprint-like HTL on its surface, improving hole extraction and transport. Consequently, the CsPbI_2_Br PSC achieved a PCE of 16.40% and exhibited good thermal stability. Meanwhile, He et al. used polyethylene-graft-maleic anhydride (PGMA) to passivate defects, regulate energy levels, and stabilize the perovskite structure (Figure 7e) [119]. The hydrogen bonds between the -CH_2_ groups in PGMA and the I^−^/Br^−^ ions in CsPbI_2_Br, along with the coordination interaction of the carbonyl group with Cs^+^/Pb^2+^, improved charge transport and collection. This passivation of defects and energy level management reduced non-radiative recombination losses. The acrylonitrile butadiene styrene (ABS), a long-chain polymer, was incorporated into CsPbI_2_Br to enhance grain quality and interfacial contact [120]. Due to the strong polarity of ABS molecules, the nitrogen atoms with lone pairs could effectively interact with Pb ions, while the benzene rings facilitated interactions with CsPbI_2_Br. Additionally, ABS suppressed halide ion migration and exhibited superior moisture resistance. Consequently, the PCE of CsPbI_2_Br PSCs improved from 11.80% to 14.27%.

In addition, other molecules are also used as additives. Phthalic imide (PTM) additives were introduced into the precursor to prepare high-quality CsPbI_2_Br perovskite films under ambient air conditions (Figure 7f,g) [113]. The C=O and N-H groups of PTM strongly coordinated with Pb^2+^ ions, forming hydrogen bonds with halide ions. This interaction created a CsBr-PTM-PbI_2_ intermediate phase that protected the film from moisture and phase transitions, requiring more energy to decompose. This slowed nucleation and promoted larger perovskite grain growth, improving the CsPbI_2_Br film’s efficiency to 13.95%. The degradation mechanism of CsPbI_2_Br perovskites was further investigated under light and oxygen exposure [121]. Tanshinone IIA was employed as a superoxide scavenger to enhance the environmental stability of the material. Specifically, superoxide species generated from the interaction between O_2_ and photoexcited electrons oxidized Pb-I bonds, leading to the formation of PbO and I_2_ while leaving behind the CsPbBr_3_ phase. However, tanshinone IIA effectively passivated defects and eliminated superoxide species, thereby significantly improving the photostability and efficiency of CsPbI_2_Br PSCs.

**Figure 8:**
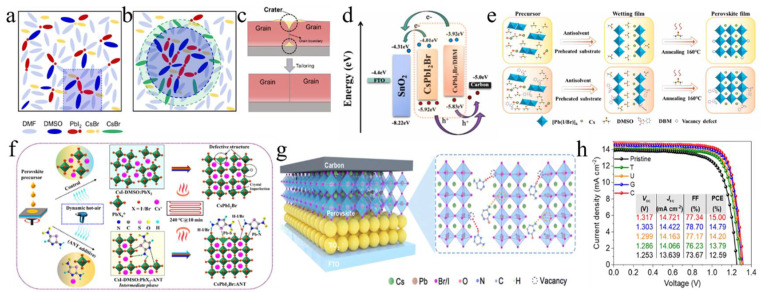
(**a**) Schematics for the conventional method. (**b**) DHBP-regulated precursors of inorganic perovskite. (**c**) Formation of craters at the grain boundary during annealing and modulated growth with hydrogen bond bridging. (**a**,**c**) Reproduced with permission [123]. Copyright 2024, American Chemical Society. (**d**) The energy level diagram. (**e**) Film-forming process diagram of CsPbI_2_Br and CsPbI_2_Br/DBM, respectively. (**d**,**e**) Reproduced with permission [124]. Copyright 2024, Elsevier. (**f**) Schematic demonstration for defect passivation via the interaction of the ANT molecule with perovskite. Reproduced with permission [126]. Copyright 2024, Wiley-VCH. (**g**) Schematic of a typical PSC and the interaction between cytosine and the perovskite film. (**h**) Characteristic J–V curves for carbon-based CsPbI_2_Br PSCs. (**g**,**h**) Reproduced with permission [125]. Copyright 2024, Royal of Society Chemistry.

Bifunctional amide molecules were incorporated into the perovskite precursor solution to regulate the crystallization process and passivate defects. By adjusting the intermediate bridging frameworks with various groups such as the alkyl, alkene, and phenyl groups, they found that the passivation strength was influenced by the electronic structure of the spin state, which affected charge distribution [122]. Notably, the phenyl-amide molecule exhibited the strongest binding with the perovskite, resulting in a significant improvement in the efficiency of carbon-based CsPbI_2_Br PSCs to 15.51% with enhanced stability. 4,4′-dihydroxybiphenyl (DHBP) was incorporated into the precursor solution (Figure 8a–c) [123]. This facilitated the formation of hydrogen bond bridges (C-N/O…H) between CsPbI_2_Br and DHBP, leading to the creation of localized high-concentration colloidal clusters at room temperature. These clusters enhanced crystal growth and phase transition, thereby improving crystallinity and ensuring complete film coverage. Moreover, coordination bonds (Pb-O/N and Pb-I/Br) contributed to the smoothing of grain boundaries and the passivation of surface defects. Consequently, a stable film with enhanced n-type characteristics was achieved, resulting in a PCE of 16.86% and a V_oc_ of 1.38 V. The film also demonstrated high tolerance to thermal heating and ultraviolet light exposure. On the other hand, dibenzoylmethane (DBM), a precursor additive with electron-rich and C=O functional groups, was introduced to regulate the crystallization process of CsPbI_2_Br perovskites (Figure 8d,e) [124]. The addition of DBM not only accelerated nucleation during perovskite crystallization but also enhanced film stability. Specifically, the C=O groups effectively passivated uncoordinated Pb^2+^ ions, shifting the Fermi level of CsPbI_2_Br and facilitating charge transfer, which in turn reduced energy losses. This modification led to a PCE of 13.46% and a V_oc_ of 1.189 V. Natural biogenetic molecules, including uracil, cytosine, guanine, and thymine, were introduced into perovskite films to enhance film growth and passivate harmful Pb^2+^ defects (Figure 8g,h) [125]. These additives also shifted the interfacial energetics towards a more n-type configuration, thereby boosting the V_bi_ in the n/n-junction. Additionally, they facilitated the formation of larger colloids, promoting heterogeneous nucleation, which slowed down crystal growth and improved long-term stability. This strategy resulted in a carbon-based CsPbI_2_Br PSC with an efficiency of 15.0%. 2-Amino-5-nitrothiazole (ANT) served as an innovative precursor additive to enhance CsPbI_2_Br film quality (Figure 8f) [126]. The -NH_2_ group in ANT coordinated with the Pb octahedra, effectively alleviating charge defects through NH=I/Br bonding. Simultaneously, the S=C-N site interacted with uncoordinated Pb^2+^ ions, reducing defect states and non-radiative recombination. This novel approach achieved an excellent device performance of 17.13% with an FF of 0.834.

## 4. Interface Engineering

In addition to optimizing the CsPbI_2_Br films, ETL, and HTL, interface engineering is an effective technique to minimize the non-radiative charge recombination of devices without breaking the characteristics of the buffer layer below or above. Furthermore, interface engineering not only regulates the growth process of perovskite crystals but also adjusts their energy level alignment, thereby significantly enhancing both the efficiency and stability of PSCs. Table 5 summarizes interface engineering and the PV performance of the corresponding PSCs.

### 4.1. The ETL/CsPbI_2_Br Interface in a Conventional n-i-p Structure

**Figure 9:**
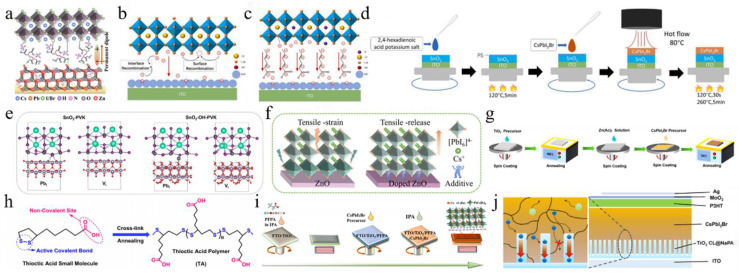
(**a**) Schematic illustration of the functionalization process of EAD. Reproduced with permission [139]. Copyright 2023, American Chemical Society. (**b**) Schematic diagram of a buried interface without PS. (**c**) Schematic diagram of the function of PS at the buried interface. (**d**) Schematic diagram of SnO_2_ layer modification and CsPbI_2_Br film preparation in air. (**b**–**d**) Reproduced with permission [145]. Copyright 2024, Elsevier. (**e**) Top view of the two types of CsPbI_2_Br perovskite bottom defects and the theoretical model of interactions between defects and hydroxyl groups. Reproduced with permission [146]. Copyright 2024, Elsevier. (**f**) Schematic illustration of the residual strain distribution of CsPbI_2_Br films on the pristine and doped ZnO ETLs. Reproduced with permission [141]. Copyright 2023, Royal of Society Chemistry. (**g**) Preparation process of the Zn(Ac)_2_-modified TiO_2_/perovskite interface. Reproduced with permission [147]. Copyright 2024, American Chemical Society. (**h**) Schematic illustration of the chemical structure of the TA small molecule, and the process of the cross-linking polymerization reaction of TA molecules after the annealing treatment. Reproduced with permission [142]. Copyright 2023, Royal of Society Chemistry. (**i**) Schematic diagram of TiO_2_ modification and CsPbI_2_Br perovskite film preparation. Reproduced with permission [148]. Copyright 2024, Elsevier. (**j**) Schematic diagram of the (**left**) electron extraction pathway from CsPbI_2_Br to the ITO electrode through TiO_2_ NaPAs and (**right**) the device architecture. Reproduced with permission [129]. Copyright 2021, Tsinghua University Press.

The interaction between SnO_2_ and CsPbI_2_Br in PSCs was modulated by EAD ZnO as an effective burial interface, resulting in a PCE of 14.58% (Figure 9a) [139]. The insertion of EAD passivated ZnO defects, regulated energy level alignment, and eliminated buried interface defects via coordination and hydrogen bonding with the CsPbI_2_Br film. This improved interface contact, released residual strain, mitigated halide ion migration, and suppressed charge recombination. Cadmium halides (CdCl_2_, CdBr_2_, and CdI_2_) were utilized to modify the SnO_2_/CsPbI_2_Br interface through a bidirectional thermal diffusion process [140]. This treatment effectively passivated defects within the SnO_2_ film and adjusted the energy level structure of the SnO_2_ ETL, thereby enhancing carrier transfer efficiency and improving hole blocking. The resulting CsPbI_2_Br films exhibited superior crystallinity, larger grain sizes, and reduced defect densities. Consequently, the CsPbI_2_Br device with CdCl_2_ achieved a PCE of 14.47%. Given that traditional interface modifiers often possess insulating properties that impede carrier transport, 2,4-hexadienoic acid potassium salt (C_6_H_7_KO_2_, PS) could be utilized as a modifier to enhance the SnO_2_/CsPbI_2_Br interface (Figure 9b–d) [145]. The conductivity of SnO_2_ was improved via the synergistic effects of K^+^ ions and conjugated groups, leading to optimal energy level alignment. Moreover, PS effectively passivated buried interface defects, promoted crystallization, enhanced film quality, and reduced non-radiative recombination. Consequently, the PCE of the PS-modified device was increased by 13.11%. Ammonium tetrafluoroborate (NH_4_BF_4_) could be utilized to modify the SnO_2_ ETL by optimizing interfacial carrier dynamics and mitigating CsPbI_2_Br defects (Figure 9e) [146]. The NH_4_^+^ ions effectively repaired hydroxyl groups on the SnO_2_ surface, thereby enhancing the energy level alignment between SnO_2_ and CsPbI_2_Br. Additionally, the BF_4_^−^ anions played a pivotal role in regulating crystal growth and minimizing defect formation. Furthermore, the removal of hydroxyl groups of the buried interface increased the activation energy required for iodide migration in CsPbI_2_Br, thereby enhancing device stability. Consequently, this approach led to optimized device performance with a PCE of up to 17.09%.

ZnO is an appealing candidate for the ETL in PSCs. However, defects at the ZnO/perovskite interface result in considerable interfacial recombination losses. To address this issue, cesium salts containing acetate (AC^−^), fluoride (F^−^), and trifluoroacetate (TFA^−^) anions were introduced to regulate ZnO deposition (Figure 9f) [141]. The multifunctional cesium modulator coordinated with Zn^2+^ and Pb^2+^ ions, effectively passivating defects and forming Zn-O-Cs interface dipoles to minimize the interfacial energy gradient. Among these anions, TFA^−^ exhibited superior performance in promoting charge extraction and transfer compared to AC^−^ and F^−^, resulting in a PCE of 14.25% for the CsTFA-modified CsPbI_2_Br device. Furthermore, cross-linked thioic acid (TA) small molecules were utilized to modify the ZnO/CsPbI_2_Br interface (Figure 9h) [142]. After heat treatment, the TA molecules formed an in situ continuous polymer network, effectively passivating surface defects, improving interfacial contact and energy level alignment, and suppressing carrier recombination. Finally, the PCE of the device was significantly enhanced to 16.56%.

On the other hand, Wang et al. introduced an equimolar mixture of TiCl_4_ and TiCl_3_ at the TiO_2_/CsPbI_2_Br interface, achieving a PCE of 14.46% [128]. This improvement could be attributed to the TiCl_4_–TiCl_3_ interface layer, which facilitated perovskite crystallization. Furthermore, the hydrolysis of Ti(III) led to a Cl-doped TiO_2_ surface, thereby enhancing interfacial electron coupling by promoting electron transfer and inhibiting charge recombination losses. In photoelectric devices, semiconductor nanocolumn arrays could reduce light reflection losses and inhibit exciton recombination dynamics. The low-temperature nanocolumn arrays (NaPAs) embedded on CsPbI_2_Br films not only improved interface contact but also facilitated electron injection and charge separation more efficiently than a dense TiO_2_ ETL (Figure 9j) [129]. As a result, the PCE of the device was improved to 11.35%. Additionally, the TiO_2_ NaPAs could guide incident light, thereby enhancing the light-trapping capability of the CsPbI_2_Br material. Recently, the deposition of Zn(Ac)_2_ onto the TiO_2_ ETL surface was achieved using a spin-coating technique (Figure 9g) [147]. The anchoring of Zn(Ac)_2_ on TiO_2_ not only passivated oxygen vacancy defects but also optimized the energy level alignment. Additionally, some AC^−^ ions may dissolve into the perovskite precursor solution, thereby inhibiting nucleation and enhancing the crystallinity and grain size of CsPbI_2_Br. Consequently, the PCE of the device achieved 14.20%. Simultaneously, perfluoropropionic acid (PFPA) could modulate both the CsPbI_2_Br/TiO_2_ and CsPbI_2_Br/carbon interfaces (Figure 9i) [148]. During the annealing process, PFPA diffused along the grain boundaries to the upper surface of the perovskite layer, thereby effectively passivating grain boundary defects and interacting with lead-related defects. This interaction inhibited non-radiative recombination and promoted the formation of an energy level gradient between the perovskite and carbon electrodes, facilitating efficient charge extraction. Moreover, the PFPA-modified TiO_2_ ETL alleviated interfacial tensile stress in the perovskite film and mitigated lattice strain. As a result, the optimized CsPbI_2_Br device achieved a PCE of 14.15%.

Borophene quantum dots (BQDs) demonstrated significant interactions with Ti^4+^ ions in TiO_2_ and Pb^2+^ ions in perovskite materials, effectively passivating the interface and reducing defect density [130]. Furthermore, an energy gradient was established at the TiO_2_/CsPbI_2_Br interface, which enhanced electron transport by forming a cascaded energy alignment and thereby suppressing carrier recombination. As a result, the device achieved a PCE of 15.31%. Given the challenges associated with growing perovskite films on perovskite quantum dot (PQD) substrates using conventional solution-based methods, a novel PQD dynamic-mediated perovskite film growth (PDMG) technique has been developed [136]. During the PDMG process, PQDs served as interface nucleation centers, thereby promoting perovskite crystallization, passivating perovskite defects, enhancing interfacial contact, suppressing TiO_2_/CsPbI_2_Br interface defects, and improving charge extraction and transport. As a result, for CsPbI_2_Br PSCs, the PCE was enhanced from 10.44% to 12.14%.

### 4.2. The CsPbI_2_Br/HTL Interface in a Conventional n-i-p Structure

**Figure 10:**
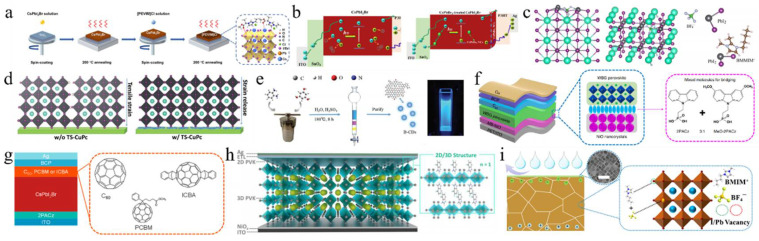
(**a**) Schematic illustration of surface modification and possible surface passivation mechanisms for CsPbI_2_Br perovskite films. Reproduced with permission [132]. Copyright 2021, Elsevier. (**b**) Schematic diagram of carrier transfer for the PSCs without and with the CsPbBr_3_ NC treatment. Reproduced with permission [100]. Copyright 2023, American Chemical Society. (**c**) Calculated structure illustrating the passivation of an I^−^ vacancy at the CsPbI_2_Br surface by a BF_4_^−^ anion. Reproduced with permission [137]. Copyright 2022, Springer Singapore. (**d**) Schematic diagram of tensile strain distribution in the CsPbI_2_Br layer without and with a TS-CuPc-i underlayer. Reproduced with permission [134]. Copyright 2021, Wiley-VCH. (**e**) Preparation method of the p-type B-CDs using the solvothermal approach. Reproduced with permission [133]. Copyright 2021, Wiley-VCH. (**f**) Device structure and molecular structures of bridging molecules. Reproduced with permission [151]. Copyright 2022, Springer Nature. (**g**) Device architecture of CsPbI_2_Br PSCs and chemical structures of ETLs. Reproduced with permission [152]. Copyright 2023, American Chemical Society. (**h**) Schematic structure of the inverted PSC with a 2D/3D hybrid structure. Reproduced with permission [135]. Copyright 2021, American Chemical Society. (**i**) TEM image showing the size distribution of the QDs. Reproduced with permission [144]. Copyright 2023, American Chemical Society.

An annealed film was subjected to post-treatment with guanidinium bromide (GABr) to induce secondary crystallization. The bromide ions from GABr diffused into the perovskite bulk phase through an ion-exchange reaction, forming a bromine-rich region [131]. This bromine-rich region functioned as an effective charge collection center, enhancing the device’s efficiency. Additionally, this treatment led to an increase in the bandgap, adjustment of the Fermi level, and improved energy band alignment with the HTL. The PCE of the modified CsPbI_2_Br device was measured at 16.97%. Ionic liquids have garnered significant attention for their notable enhancement of PSC properties. The ionic liquid 1-vinyl-3-propylammonium ethyl imidazolium chloride ([PEVIM]Cl) was used to modify the surface of CsPbI_2_Br perovskite films (Figure 10a) [132]. [PEVIM]Cl exhibited strong interactions with undercoordinated Pb and Cs metal ions, effectively passivating surface trap states, reducing non-radiative recombination, and enhancing charge transport. Consequently, the PCE of the device reached 14.19%, demonstrating excellent thermal and moisture stability. Furthermore, uncoordinated Pb^2+^ ions could form coordination bonds with the C-N and C=N functional groups in 1-butyl-2,3-dimethylimidazolium tetrafluoroborate (BMMIMBF_4_) (Figure 10c) [137]. The BF_4_^−^ anions effectively passivated Pb^2+^ and Cs^+^ ions through the formation of ionic bonds, thereby reducing interfacial non-radiative recombination and optimizing the energy level alignment at the CsPbI_2_Br/Spiro-OMeTAD interface. This enhancement facilitated hole transport and minimized interfacial recombination. Surface modification of CsPbI_2_Br PSCs resulted in a PCE of 17.02%. Carbon dots, a type of carbon nanomaterial, have garnered significant attention due to their exceptional properties. Blue carbon dots (B-CDs) exhibited a rich array of functional groups that enable effective interaction with perovskite ions through hydrogen and coordination bonds, effectively passivating defects (Figure 10e) [133]. The prepared B-CDs exhibited p-type semiconductors, forming a P–N junction with n-type CsPbI_2_Br perovskite, thereby facilitating hole transfer and inhibiting electron flow. Moreover, the incorporation of B-CDs enhanced the hydrophobicity of the perovskite film, significantly improving its stability. Utilizing p-type B-CDs (with an approximate size of 10 nm) as a surface modification layer for CsPbI_2_Br PSCs resulted in a PCE of up to 16.76%.

A dual-functional strategy incorporating CsPbBr_3_ nanocrystals (NCs) was employed to passivate bulk and surface defects within the CsPbI_2_Br layer (Figure 10b) [100]. The introduction of CsPbBr_3_ NCs facilitated the formation of an “electronic bridge”, which enhanced electron transport efficiency. Furthermore, the CsPbBr_3_ NCs modified the surface, establishing a gradient heterojunction between the CsPbI_2_Br and P3HT layers. Consequently, this approach led to a significant enhancement in the PCE of CsPbI_2_Br PSCs, achieving a PCE of 17.03%.

### 4.3. The HTL/CsPbI_2_Br Interface in an Inverted p-i-n Structure

In light of the challenges associated with forming continuous films on the surface of PTAA layers and the low light transmittance of p-type Spiro-OMeTAD films, a hydrophilic undoped Spiro-OMeTAD:PTAA mixed layer (SpiPA-II) was synthesized [153]. Subsequently, this hybrid film was employed as the HTL to fabricate an inverted PSC with the structure ITO/SpiPA-II/CsPbI_2_Br/ZnO:C_60_/Ag, achieving a PCE of 12.52%. Compared to single-component films, SpiPA-II not only promoted the growth of large-grain CsPbI_2_Br perovskite crystals but also reduced the defect density, thereby enhancing the device’s performance. A Spiro-OMeTAD super-film modified with copper phthalocyanine sodium tetrasulfate (TS-CuPc) was developed as an HTL to address the common interface stress issue at the HTL/CsPbI_2_Br interface (Figure 10d) [134]. The TS-CuPc layer formed strong coordination bonds with the perovskite and exhibited weak van der Waals interactions with Spiro-OMeTAD. This imbalance in interactions resulted in the TS-CuPc/CsPbI_2_Br film contracting towards Spiro-OMeTAD during the cooling phase post-thermal annealing, thereby alleviating interfacial tensile strain. Additionally, the TS-CuPc layer facilitated Pb-O coordination and electrostatic interactions with the perovskite, achieving effective interface passivation. This reduced interface defects and enhanced hole extraction and charge transport efficiency. Finally, the PCE of the CsPbI_2_Br device was 14.85%.

Low-temperature processed NiO_x_ has garnered significant attention owing to its excellent chemical stability, high optical transparency, and cost-effective manufacturing. However, the performance of PSCs is limited by the high density of traps and the misalignment of energy levels at the NiO_x_/perovskite interface. Surface modification with self-assembling materials (SAMs) is an effective way to improve the surface properties and the PCE of PSCs. For example, two molecules featuring a carbazole core and phosphate anchoring groups were utilized to self-assemble into a monolayer interfacing with the low-temperature-treated NiO nanocrystal film, thereby forming a bridging perovskite structure (Figure 10f) [151]. Owing to enhanced energy level alignment, reduced interfacial recombination, and improved hole extraction enabled by the molecule-bridged interface, the flexible wide-bandgap CsPbI_2_Br PSCs exhibited a substantial increase in PCE from 13.5% to 16.2%. Two SAMs, namely [2-(9H-carbazol-9-yl)ethyl] phosphonic acid (2PACz) and 2-(3,6-dimethoxy-9H-carbazol-9-yl)ethyl phosphonic acid (MeO2PACz), were used as the HTL (Figure 10g) [152]. It is worth noting that the quasi-Fermi level splitting (QFLS) values of the CsPbI_2_Br films deposited on ITO/MeO-2PACz (1.47 eV) or ITO/2PACz (1.50 eV) were almost the same as those of the CsPbI_2_Br films deposited on glass/CsPbI_2_Br (1.51 eV), which indicated that the HTL/CsPbI_2_Br interface had excellent passivation effects.

The development of a hydrophilic Spiro-OMeTAD:PTAA hybrid HTL and strain-relieving TS-CuPc interlayers has significantly improved hole extraction in inverted p-i-n devices. While these methods enhance interfacial contact and reduce recombination, their dependence on costly organic HTMs such as Spiro-OMeTAD raises concerns about scalability and cost-effectiveness. Inorganic alternatives (e.g., NiO_x_ with SAMs) offer better stability but suffer from lower hole mobility. Future research should prioritize low-cost, dopant-free polymers or inorganic/organic hybrids to bridge this gap.

### 4.4. The CsPbI_2_Br/ETL Interface in an Inverted p-i-n Structure

Unlike conventional heterostructures that require stringent lattice matching to minimize interfacial defects and strain-induced deformations, 2D perovskite layers exhibit exceptional compatibility with diverse substrates due to their van der Waals interactions. This unique characteristic eliminates the need for epitaxial alignment, thereby simplifying fabrication processes while significantly reducing interfacial defects and lattice mismatches. The inherent flexibility of 2D materials enhances charge carrier mobility by mitigating trap states at interfaces, as demonstrated in prior studies on 2D layered systems [154]. In situ 2D/3D hybrid heterojunction perovskites have garnered significant attention due to their integration of the advantageous properties of both 2D and 3D perovskites. The 2D/3D hybrid structure was synthesized through the surface treatment of CsPbI_2_Br films with benzimidazolium iodide (BIZI) (Figure 10h) [135]. This approach not only enhanced the material’s moisture resistance but also significantly passivated defects, effectively blocking holes at the CsPbI_2_Br/PC_61_BM interface. The device treated with BIZI achieved a PCE of 14.32% and demonstrated excellent stability. Liu’s group incorporated a variety of organic cations, including phenylmethylammonium iodide, 4-trifluoromethylphenylmethylammonium iodide, phenylethylammonium iodide with different side groups (-OCH_3_ and -CH_3_), 2-thiophenemethylammonium iodide (TM), 2-thiopheneethylammonium iodide, and benzenium iodides with different side groups (-H and -CF_3_), into the surface of CsPbI_2_Br perovskite films [149,150,155,156,157]. This approach not only passivated surface defects and prolonged the carrier lifetime but also inhibited non-radiative recombination. Additionally, it optimized the energy level alignment at the CsPbI_2_Br/PCBM interface, thereby enhancing charge transfer from the perovskite to the ETL. Consequently, the performance of CsPbI_2_Br PSCs was significantly improved. Specifically, the PCE of the device (ITO/NiO_x_/CsPbI_2_Br/TM/PCBM/BCP/Ag) treated with TM reached 15.07%.

A dual-sided healing strategy was proposed to concurrently enhance the performance of both perovskites and the ETL [138]. Bipyrimidine hydroiodide (BP-HI) diffused into the perovskite grain boundaries (GBs) and the ZnO layer, effectively passivating defects. This led to an increase in quasi-Fermi level splitting, enhancement of the V_bi_, and improvement in interface contact. The dual-sided passivation approach not only improved charge conduction but also inhibited ion migration. After BP-HI treatment, the PCE of the CsPbI_2_Br device reached 15.36%. In a follow-up study, an efficient CsPbI_2_Br/TiO_2_ heterostructure was constructed by incorporating TiO_2_ nanoparticles (NPs) into the chlorobenzene antisolvent, thereby concurrently depositing the CsPbI_2_Br perovskite layer and the top TiO_2_ ETL [143]. The TiO_2_ NPs facilitated the regulation of perovskite nucleation and growth via Pb-O and I/Br-Ti bonding templates during the crystallization process. This ensured intimate contact at the heterojunction, enhanced interfacial quality, and suppressed non-radiative recombination. As a result, the PCE of the fabricated device achieved 17.1%. Furthermore, CsPbI_2_Br films were modified through the incorporation of 1-butyl-3-methylimidazolium tetrafluoroborate (BMIMBF4) ionic liquid (IL) and CsPbBr_3_ QDs (Figure 10i) [144]. The IL significantly influenced the orientation of grain growth, resulting in a decrease in defect density, an increase in grain size, and enhanced light absorption. The QD layer offered protection against water molecule-induced degradation, thereby improving structural stability. Consequently, the PCE of the CsPbI_2_Br-IL/QD PSCs attained 15.37%.

Interface engineering plays a pivotal role in enhancing the efficiency and stability of PSCs by finely tuning the interactions between different layers, passivating defects, adjusting energy levels, and improving charge transfer and transport properties.

## 5. Charge Transport Layers

### 5.1. Electron Transport Layers

ETLs play a crucial role in PSCs by facilitating the transport and collection of electrons, which substantially influence the photoelectric performance and stability of the devices. Key characteristics of ETLs, such as electron mobility, work function, and chemical stability, significantly impact the efficiency and lifetime of PSCs. Consequently, optimizing the architecture and properties of ETLs is a critical strategy for enhancing the performance of these solar cells. Table 6 summarizes CTL strategies and the PV performance of the corresponding PSCs.

**Figure 11:**
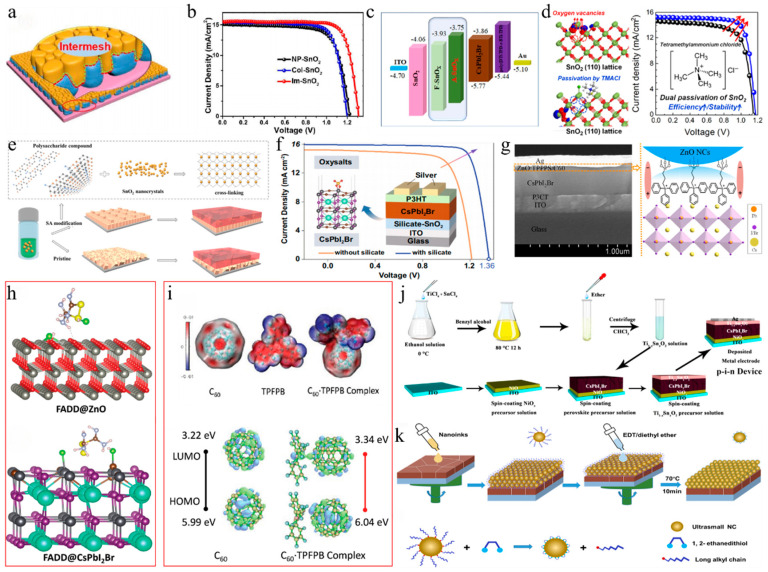
(**a**) Schematic illustration of the combination process of NP-SnO_2_ and Col-SnO_2_ via the intermeshing SnO_2_ ETL strategy. (**b**) J–V curves of PSCs with different SnO_2_. (**a**,**b**) Reproduced with permission [162]. Copyright 2020, Royal of Society Chemistry. (**c**) Energy level diagrams of the components in the device. Reproduced with permission [161]. Copyright 2020, American Chemical Society. (**d**) Iso-surface plots of the highest occupied valence band and the lowest occupied conduction band of SnO_2_ (110) with oxygen vacancies and J–V curves of devices. Reproduced with permission [166]. Copyright 2021, American Chemical Society. (**e**) Schematic illustration of SnO_2_ with SA. Reproduced with permission [183]. Copyright 2024, Wiley-VCH. (**f**) Optimized structure of the CsPbI_2_Br (001) surface and J–V curves of PSCs without silicate and with silicate. Reproduced with permission [167]. Copyright 2021, Springer Singapore. (**g**) The cross-sectional SEM image of the inverted CsPbI_2_Br PSCs (**left**), and schematic illustration of the CsPbI_2_Br/ZnO:TPPPS interface (**right**). Reproduced with permission [165]. Copyright 2021, Elsevier. (**h**) DFT-optimized models of FADD@ZnO and FADD@CsPbI_2_Br. Reproduced with permission [184]. Copyright 2024, Wiley-VCH. (**i**) The molecular structures and calculated ESP images of C_60_, TPFPB, and the C_60_·TPFPB complex, and the optimized molecular orbital graphs of C_60_ and the C_60_·TPFPB complex. Reproduced with permission [160]. Copyright 2020, Wiley-VCH. (**j**) Synthesis of ultrathin Ti_1−x_Sn_x_O_2_ NCs and fabrication of an inverted p-i-n PSC. Reproduced with permission [172]. Copyright 2022, Elsevier. (**k**) Schematic illustration of the fabrication process of efficient inorganic ETLs on top of perovskite layers using ligand exchange at low temperatures and reaction schemes for the exchange of long alkyl chains through EDT on the surface of NCs. Reproduced with permission [173]. Copyright 2022, Wiley-VCH.

Li et al. ingeniously constructed an ETL with an interconnected SnO_2_ morphology by sequentially spin-coating two SnO_2_ precursor solutions (NP-SnO_2_ and Col-SnO_2_) (Figure 11a,b) [162]. This interwoven continuous structure facilitated effective electron transfer from CsPbI_2_Br to the indium tin oxide (ITO) electrode. Owing to the high electron mobility of the Im-SnO_2_ ETL and the excellent electronic coupling between the two SnO_2_ layers, which formed a cascaded energy level, it enhanced rapid electron transport within the ETL and minimized charge recombination, thus improving charge extraction efficiency and contributing to the enhancement of solar cell performance. To enhance the electronic transport properties of the SnO_2_ ETL, an aged SnCl_2_ solution was applied to the surface of commercial SnO_2_ nanocrystals (Figure 11c) [161]. This process resulted in the formation of a uniform and smooth amorphous SnO_x_ film. The formation of this film elevated the conduction band of SnO_x_ and reduced the interfacial trap state densities within the device, leading to a maximum V_oc_ of 1.43 V and a PCE of 15.53%. Furthermore, a potassium fluoride (KF)-doped SnO_2_ ETL was synthesized, demonstrating enhanced compatibility with the conduction band of the CsPbI_2_Br layer [168]. Concurrently, F^−^ ions were capable of migrating into the perovskite film, effectively reducing the non-radiative recombination and trap density within the perovskite film. Experimental results indicated a significant enhancement in the PCE of fluorine-doped SnO_2_-based CsPbI_2_Br PSCs, increasing from 13.40% to 15.39%. Additionally, the V_oc_ has been elevated by 130 meV. Tetramethylammonium chloride (TMACl) was incorporated into the SnO_2_ solution, thereby enhancing the charge transfer and extraction performance via dual passivation of oxygen vacancies (Figure 11d) [166]. This process shifted the energy level of SnO_2_ upwards, leading to improved energy level alignment with CsPbI_2_Br. As a result, the SnO_2_/CsPbI_2_Br interface demonstrated efficient electron transfer and minimal charge recombination, achieving a PCE of 13.84% and improved stability. The method of using urea (CO(NH_2_)_2_) to prepare SnO_2_ colloidal NCs has been developed to control the formation kinetics of SnO_2_ nanocrystals [179]. The prepared SnO_2_ NCs exhibited superior electrical conductivity, high electron mobility, low trap density, and enhanced energy level alignment, which significantly enhanced charge extraction and suppressed non-radiative recombination in PSCs. Ultimately, the CsPbI_2_Br PSC achieved a V_oc_ of 1.30 V and a PCE of 16.22%. Incorporating sodium alginate (SA) into the SnO_2_ colloidal solution formed a nanostructured synapse, aligned the energy levels, inhibited charge recombination, and significantly enhanced the extraction and transport of carriers (Figure 11e) [183]. Additionally, the coordination bonds formed between the Sn atoms and the carboxyl ions derived from SA could reduce defects at the SnO_2_/perovskite interface. The PCE of the CsPbI_2_Br solar cell reached 16.90%. Oxoanions were added to the SnO_2_ colloidal aqueous solution to modify the SnO_2_ film (Figure 11f) [167]. This modification optimized the energy band structure at the SnO_2_/perovskite interface, which facilitated electron transport. Consequently, the CsPbI_2_Br PSCs achieved a PCE of 17.26% and demonstrated excellent device stability.

Cesium acetate (CsAc) and lead acetate (Pb(Ac)_2_) dopants were chemically doped in situ into the ZnO ETL, shifting its energy levels upwards to achieve optimal alignment with those of CsPbI_2_Br [185]. This enhanced alignment, in conjunction with other factors, reduced interfacial energy loss, leading to a V_oc_ of 1.282 V and a champion PCE of 16.36% for the CsPbI_2_Br PSC. Aluminum-doped ZnO (AZO) was employed as the ETL, exhibiting favorable energy level alignment with the CsPbI_2_Br perovskite [163]. Ultimately, the optimized CsPbI_2_Br PSCs attained a PCE of 15.08%. The incorporation of 3-triphenylphosphonylpropane-1-sulfonate (TPPPS) into the ZnO precursor solution effectively passivated surface defects on the ZnO and at the CsPbI_2_Br/ZnO interface (Figure 11g) [165]. Simultaneously, this treatment facilitated the formation of an optimal energy level alignment at the cathode contact, leading to more efficient electron extraction from the perovskite layer to the cathode. Consequently, the champion device efficiency was significantly improved to 14.62%. In situ chemical doping of alkaline metal ions into the ZnO ETL was achieved via the sol-gel method [177]. Doping Ca^2+^ into the ZnO ETL not only enhanced the electron transfer of CsPbI_2_Br perovskite by optimizing the energy level alignment but also improved the crystallization of CsPbI_2_Br perovskite by decreasing the defect density. Consequently, the optimized ZnO:Ca-based PSC exhibited a PCE of 16.39% and a V_oc_ of 1.292 V. Dimethylammonium disulfide (DMDS) was incorporated into the ZnO ETL. This compound exhibited mechanical “softness” and offered numerous passivation sites, effectively mitigating oxygen vacancy defects in ZnO and perovskite defects, including undercoordinated Pb^2+^ and Pb^0^/I^0^ (Figure 11h) [184]. Consequently, this significantly reduced charge recombination in PSCs, leading to a PCE of 16.05%. The ZnO@C_60_ ETL structure was constructed using a co-doping strategy with tris(pentafluorophenyl)borane (TPFPB) and a non-hygroscopic lithium salt (LiClO_4_) (Figure 11i) [160]. The coordination interaction between TPFPB and C_60_ lowered the lowest unoccupied molecular orbital (LUMO) energy level of C_60_, thereby enhancing the electron extraction efficiency and reducing the electron trap density. Additionally, the addition of the non-hygroscopic LiClO_4_ improved the electron mobility and conductivity of the film, effectively mitigating the hysteresis phenomenon. Consequently, the CsPbI_2_Br PSC achieved a maximum PCE of 15.19% and a stable power output (SPO) of 14.21%.

Uniformly depositing oleic acid (OA)-capped monodisperse metal oxide (MOX) NCs has been proposed to achieve a flat, uniform, and needle-free state of the hybrid C-TiO_2_ ETL [158]. This method regulated the conductivity and energy band structure of the ETL. Consequently, the CsPbI_2_Br PSC achieved a PCE of 14.0%. A non-hydrolytic sol-gel method was developed to synthesize Sn-doped TiO_2_ (Ti_1−x_Sn_x_O_2_) NCs, which enhanced the electrical conductivity of Ti_0.9_Sn_0.1_O_2_ and optimized the energy level alignment between the energy level of the conduction band (CB) and the perovskite active layer (Figure 11j) [172]. Additionally, the minor presence of residual Cl^−^ ions occupied the oxygen vacancies, effectively passivating the I^−^ defects in the perovskite film, thus reducing non-radiative recombination. As a result, a PCE of 15.18% was achieved for CsPbI_2_Br PSCs. Multi-contact ETLs, designated as TiO_2_@Sb_2_S_3_-MPA ETLs, were prepared using the thermal injection method [178]. The TiO_2_@Sb_2_S_3_-MPA ETL enhanced electrical conductivity, optimized charge transfer, and mitigated interfacial recombination with the CsPbI_2_Br film. Consequently, the PCE of the CsPbI_2_Br PSC reached up to 14.59%.

Atomic layer deposition (ALD) was employed to fabricate In_2_O_3_ films, which exhibited polycrystalline cubic structures and demonstrated superior optical and electrical properties [186]. Upon integration into the device, the In_2_O_3_ ETL-based CsPbI_2_Br PSC achieved a PCE of 10.97%. Low-temperature liquid-treated metal sulfide ETLs were prepared via a ligand-exchange process in an anhydrous ether solution containing 1,2-ethylenedithiol (EDT) (Figure 11k) [173]. These ETLs exhibited smooth, dense, and pinhole-free morphologies that are well-matched with CsPbI_2_Br, demonstrating excellent electrical conductivity and chemical stability. Consequently, the device attained a PCE of 15.04%.

ETLs play a pivotal role in determining the performance and stability of CsPbI_2_Br PSCs. Various strategies have been employed to optimize ETLs, which not only enhance electron mobility but also improve energy level alignment and mitigate interfacial defects. Consequently, these optimizations result in substantial improvements in PCE and V_oc_. Advancements in the design and modification of ETLs are indispensable for the ongoing progress and refinement of PSC technology.

### 5.2. Hole Transport Layers

CsPbX_3_ perovskites have attracted widespread attention for their excellent thermal stability, but their sensitivity to humidity remains a major challenge. Common HTLs, such as Spiro-OMeTAD, typically require the addition of hygroscopic dopants (such as lithium salts, cobalt salts, and 4-tert-butylpyridine (TBP)) to improve charge carrier conductivity. However, these traditional hygroscopic dopants are prone to inducing phase transitions in CsPbI_2_Br perovskites, making them unsuitable for these materials. Moreover, an undoped HTL suffers from significant electrical losses, making it difficult to achieve an efficient PCE. To address these issues, researchers have developed a variety of organic molecules and polymers as new HTLs. For example, P3HT was deposited on the top of the perovskite film to achieve the effective passivation of defect states [18]. This material exhibited efficient hole extraction capabilities owing to its favorable energy level alignment and strong interaction with CsPbI_2_Br. The device achieved a PCE of 12.02% and a V_oc_ of 1.32 V.

**Figure 12:**
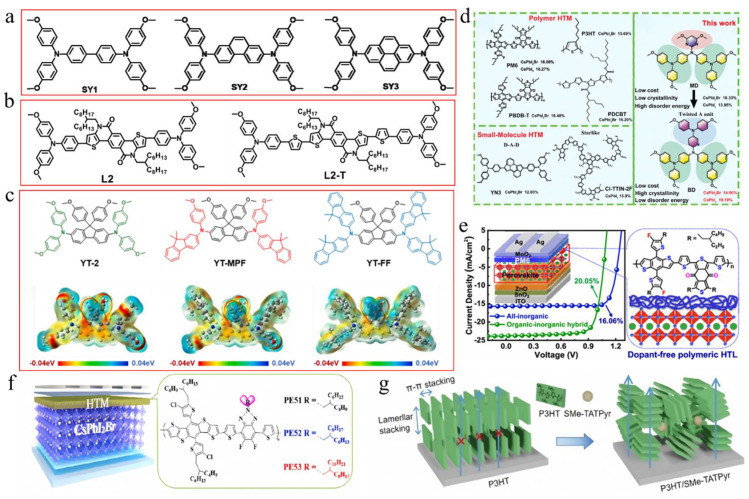
(**a**) Molecular structures of SY1, SY2, and SY3. Reproduced with permission [164]. Copyright 2021, Royal of Society Chemistry. (**b**) Molecular structures of L2 and L2-T. Reproduced with permission [187]. Copyright 2021, Elsevier. (**c**) Molecular structures of YT-2, YT-MPF, and YT-FF, and electrostatic surface potentials of YT-2, YT-MPF, and YT-FF. Reproduced with permission [188]. Copyright 2021, Elsevier. (**d**) Some dopant-free HTMs used in PSCs and the molecular structure of dopant-free MDs and BD HTMs for PSCs. Reproduced with permission [189]. Copyright 2024, Wiley-VCH. (**e**) Device structure and J–V curves of CsPbI_2_Br PSCs. Reproduced with permission [171]. Copyright 2021, American Chemical Society. (**f**) Device structure of CsPbI_2_Br PSCs and the molecule structure of PE51, PE52, and PE53. Reproduced with permission [180]. Copyright 2023, Elsevier. (**g**) Schematic diagram of the molecular stacking. Reproduced with permission [169]. Copyright 2021, Wiley-VCH.

**Organic small molecules:** D-π-D (donor-π-donor) materials demonstrate superior hole-transporting properties in the field of organic photovoltaics. Three cost-effective D-π-D hole transport materials (HTMs) with different π-bridge groups, including biphenyl (SY1), phenanthrene (SY2), and pyrene (SY3), were synthesized via a one-pot reaction and subsequently employed to replace the Spiro-OMeTAD HTL (Figure 12a) [164]. Among these, HTM SY3, featuring pyrene as the π-bridge, exhibited higher hole mobility, enhanced hole extraction/transport abilities, and superior film-forming properties compared to the other two HTMs. The device incorporating SY3 as the HTM achieved a PCE of 13.41% in the CsPbI_2_Br PSCs. Subsequently, two novel undoped D-A-D-type HTMs (L2 and L2-t) were designed and synthesized utilizing a ladder-shaped TPTI as the acceptor unit and TPA groups as the donor unit (Figure 12b) [187]. CsPbI_2_Br devices with L2 yielded a PCE of 12.41%, which is attributed to their favorable energy level alignment, enhanced hole mobility, and smooth film morphology. Then, two cost-effective fluorene-terminal-modified HTMs (YT-MPF and YT-FF) were designed and synthesized (Figure 12c) [188]. YT-MPF featured a single peripheral fluorene arm on either side, resulting in a more polarized structure that facilitated intermolecular interactions and enhanced hole-hopping transport. Upon incorporation into CsPbI_2_Br PSCs, YT-MPF yielded a relatively high PCE of 16.0% and a V_oc_ of 1.29 V. This performance surpassed that of control devices utilizing conventional HTMs such as PTAA (12.7%) and Spiro-OMeTAD (14.7%). Recently, two undoped star-shaped molecules (BD and MD) have been synthesized as alternatives to doped Spiro-OMeTAD (Figure 12d) [189]. In comparison to the disordered stacking of MD, the undoped BD hole-HTM featured distorted acceptor units and strong dipoles, which facilitated the formation of a crystalline and well-ordered stacked film. This enhanced charge transfer within the junction and increased the transfer rate. CsPbI_2_Br PSCs with BD achieved a PCE of 14.96%. Additionally, sequential sub-stacking effectively blocked the migration pathway of I^−^ ions to the gold electrode, thereby enhancing the stability of the device.

**Organic polymers:** The polymer donor material (PDM) PDCBT with a deep HOMO level was employed as the undoped HTL in CsPbI_2_Br PSCs [159]. The HOMO level of PDCBT aligned well with the valence-band top energy level of CsPbI_2_Br (−6.08 eV), facilitating efficient hole extraction from the perovskite layer to the anode and minimizing energy loss during the hole transfer process. Ultimately, a PCE of 16.2% was achieved for the CsPbI_2_Br PSCs. A new polymer, poly(3-dodecylthiophene-2,5-diyl-alt-2,1,3-benzothiadiazole-4,7-diyl) (PDTDT), was synthesized through specific copolymerization reactions to replace P3HT and exhibited a high hole mobility of 3.14 × 10^−3^ cm^2^/Vs with a face-on molecular orientation and an appropriate HOMO level (−5.44 eV) [170]. Through the interaction between the carbonyl group in the perovskite and the Pb atom, this polymer could effectively passivate surface traps, reduce interface trap density, and inhibit carrier recombination, thereby enhancing hole extraction. The CsPbI_2_Br PSCs attained an efficiency of 17.36% (V_oc_ = 1.42 V, FF = 0.812). Moreover, the fluorine-substituted polymer donor material, PM6, was employed as the undoped HTM (Figure 12e) [171]. This material demonstrated well-aligned energy levels, high charge carrier mobility, and efficient defect passivation. The CsPbI_2_Br PSCs with PM6 achieved a PCE of 16.06%. Li et al. introduced a one-source strategy, utilizing the same PDM as both the dopant and the HTL in CsPbI_2_Br PSCs [95]. The PDM functioned as a soft template to facilitate the growth of high-quality CsPbI_2_Br crystals and induced a downward shift in the Fermi level (E_F_) of CsPbI_2_Br, thereby optimizing the energy level alignment within the device. Additionally, the PDM formed an ultrathin fibrous film on the top surface of CsPbI_2_Br, improving the electrical contact between the layers. Consequently, CsPbI_2_Br PSCs with PBDB-T acquired a PCE of 16.40% and demonstrated robust stability.

Poly[(9-alkyl-9H-carbazole-2,7-diyl)-co-(2,4-dimethylaniline-N,N-diyl)] (PCDA), including PCDA1 (with a linear hexyl side chain) and PCDA2 (with a branched 2-ethylhexyl side chain), was synthesized through the copolymerization of a carbazole segment with strong electron-donating properties and 2,4-dimethylaniline [174]. The PCDA1 reduced the formation of pinholes in the HTL, enhanced the moisture resistance of the CsPbI_2_Br film, and optimized the energy band alignment. Consequently, the CsPbI_2_Br device with PCDA1 obtained a PCE of 11.01%. Subsequently, a nickel-based polymer HTL composed of bis(4-dimethylaminodithiophene)nickel(II) (BDMA) was developed [175]. This material effectively minimized the energy barrier at the CsPbI_2_Br/HTL interface, enhanced hole transport efficiency, and mitigated interfacial recombination. The device exhibited a PCE of 12.32%. The undoped and cost-effective polymer PTQ10 was employed as the HTL in PSCs [176]. This material demonstrated favorable energy level alignment with CsPbI_2_Br compared to the conventional P3HT. Moreover, the functional groups of PTQ10 offered effective surface passivation for the perovskite layer. As a result, the CsPbI_2_Br PSC featuring the PTQ10 HTL attained a PCE of 17.8% and a high V_oc_ of 1.4 V. The same D unit (BDT) and π bridge (thiophene), along with different A units (Qx, BDD, and BTA), were utilized to synthesize three D-π-A-type polymers as HTMs for CsPbI_2_Br PSCs [181]. Among these, PE61 exhibited superior charge transfer performance from CsPbI_2_Br, leading to a champion PCE of 16.72% for the solar cells. These findings suggested that Qx, when employed as the A unit in D-π-A polymers, was an excellent choice for the preparation of high-performance undoped HTMs. Similarly, P-BTA-xF (P-BTA-2F and P-BTA-4F) polymers with a D-π-A structure were synthesized through the fluorine substitution strategy [190]. As the degree of fluorine substitution increased, the HOMO energy level of the HTM polymer progressively shifted downward, while the surface packing density and crystallinity of the HTM were improved. Ultimately, the CsPbI_2_Br PSC incorporating the P-BTA-4F HTM gained a PCE of 17.68%.

A class of p-type conjugated polymers, specifically D18 and its derivative D18-Cl, were utilized as undoped HTLs in CsPbI_2_Br PSCs [182]. The thermal expansion coefficients of D18 and D18-Cl exceeded those of the perovskite films, thereby alleviating residual stress within the films. Consequently, the efficiency of a CsPbI_2_Br PSC with D18-Cl reached 16.73%, with an FF surpassing 0.85. In addition, undoped poly(3-alkylthiophene) (P3AT), poly(3-butylthiophene) (P3BT), poly(3-hexylthiophene) (P3HT), and poly(3-octylthiophene) (P3OT) were utilized as HTLs to investigate the compatibility issue between the alkyl chain length and the alkylammonium salt at the interface of the perovskite layer [191]. Owing to its higher sequential edge orientation and shorter lamellar spacing, P3BT, which has the shortest side alkyl chain, demonstrated superior hole extraction and transport properties. Ultimately, the combination of undoped P3BT as the HTL with butylammonium (nBABr) interface passivation enhanced the performance of CsPbI_2_Br PSCs. Similarly, a series of D-π-A-type polymeric materials with alkyl side chains of varying lengths (PE51, PE52, and PE53) were systematically designed, incorporating dithieno [2,3-d;2′,3′-d’]benzo [1,2-b;4,5-b’]dithiophene (DTBDT) as the donor unit, benzo[d][1,2,3]triazole (BTA) as the acceptor segment, and thiophene as the π-bridge (Figure 12f) [180]. As the alkyl chain length increased from 2-butyloctyl (PE51) to 2-hexyldecyl (PE52) and 2-octyldodecyl (PE53), the HOMO energy level of the polymers progressively decreased, and the molecular stacking transitioned from edge-on to face-on. Furthermore, among the three polymers, PE53 displayed the most effective hole extraction at the CsPbI_2_Br/HTL interface. Consequently, the CsPbI_2_Br PSC employing PE53 as the HTL yielded the highest PCE of 17.65%.

**Doping agent engineering:** The donor-type small molecule SMe-TATPyr was introduced into the P3HT solution to modulate the stacking characteristics of P3HT (Figure 12g) [169]. The addition of SMe-TATPyr disrupted the long-range ordered “edge-on” structure of P3HT, promoting the formation of “face-on” vertically π-π-stacked P3HT clusters, which enhanced efficient carrier transport in vertically structured solar cells and improved hole mobility. Furthermore, the energy levels of the P3HT/SMe-TATPyr were well-matched with those of CsPbI_2_Br, resulting in a reduction of 190 meV in energy loss compared to pure P3HT. Ultimately, the CsPbI_2_Br PSC incorporating P3HT/SMe-TATPyr attained a PCE of 16.93% and demonstrated superior moisture and heat resistance. Furthermore, polyvinylcarbazole (PVK) was incorporated into the PTAA precursor solution, thereby minimizing the energy offset between the valence band maximum (VBM) of CsPbI_2_Br and PTAA [192]. This enhancement facilitated more efficient hole extraction and effectively mitigated charge recombination within the CsPbI_2_Br bulk film, as well as at the CsPbI_2_Br/PTAA interface. Consequently, the CsPbI_2_Br device yielded a PCE of 13.60%.

Advancements in the development of organic molecules and polymers as HTLs have substantially enhanced the performance of CsPbX_3_ PSCs, effectively mitigating the challenges associated with traditional hygroscopic dopants. By employing innovative materials and engineering strategies, researchers have achieved significant efficiency improvements, thereby highlighting the promising potential of these alternative HTLs in advancing PSC technology.

Undoped polymeric HTLs such as PDTDT and PM6 represent a paradigm shift in CsPbI_2_Br PSCs, achieving PCEs > 17% while avoiding hygroscopic additives. Their tunable energy levels and defect-passivating functional groups (e.g., carbonyl–Pb interactions) are significant strengths. However, batch-to-batch variability in polymer molecular weight and crystallinity poses challenges for reproducibility. Scalable synthesis techniques, such as flow chemistry, could standardize polymer quality and accelerate commercialization.

## 6. Challenges and Outlooks for CsPbI_2_Br PSCs

Despite the remarkable advancements in CsPbI_2_Br PSCs, several critical challenges remain to be addressed for their widespread commercialization. One significant challenge lies in the phase instability of CsPbI_2_Br under ambient conditions. Black cubic α-phase CsPbI_2_Br, which is essential for efficient PSC performance, tends to convert into the more stable orthorhombic δ phase at room temperature, compromising the device’s efficiency and stability. Additionally, halide segregation, where iodide and bromide ions migrate through halogen vacancies, can lead to the formation of I-rich and Br-rich domains, further exacerbating phase instability. Another significant challenge is the energy loss within CsPbI_2_Br PSCs, which can primarily be attributed to the inferior film quality derived from solution-processing techniques, imperfect energy band offsets at the interfaces, and thermal expansion mismatches between the perovskite film and carrier transport layers. These factors result in severe non-radiative charge recombination and a consequent reduction in the V_oc_. Moreover, the high sintering temperature required to form high-quality CsPbI_2_Br films (typically above 300 °C) poses a significant hurdle for the fabrication of flexible and large-area devices. This high-temperature process not only consumes a substantial amount of energy but also limits the choice of substrates.

To overcome these challenges, several strategies have been proposed. One approach focuses on optimizing the CsPbI_2_Br film’s morphology and suppressing defect states through crystallization regulation and additive engineering. For instance, the incorporation of organic and inorganic additives, such as methylamine gas, ethylammonium bromide, and polysulphides, has demonstrated significant improvements in the crystallinity and phase stability of CsPbI_2_Br films. Another promising direction is the development of novel CTLs with better energy level alignment and higher carrier mobility. By designing new functional layers or doping existing layers, the energy barriers at the interfaces can be minimized, facilitating charge extraction and reducing recombination losses.

Interface engineering also plays a crucial role in enhancing the performance and stability of CsPbI_2_Br PSCs. By introducing interfacial layers that can passivate defects, optimize energy level alignment, and block moisture ingress, the device’s operational lifetime and efficiency can be significantly improved. Finally, the integration of CsPbI_2_Br PSCs into tandem solar cell architectures offers a pathway to surpass the Shockley–Queisser limit for single-junction solar cells. By combining CsPbI_2_Br with other photovoltaic materials, such as silicon or organic semiconductors, higher power conversion efficiencies can be achieved. However, further research is needed to optimize the performance of CsPbI_2_Br-based top cells in tandem devices.

## 7. Conclusions

Significant progress has been achieved in the development of CsPbI_2_Br PSCs, primarily attributed to advancements in film deposition methodologies, precise crystallization control, sophisticated interface engineering, and the optimized design of CTLs. High-temperature processing is essential for obtaining phase-pure α-CsPbI_2_Br films with superior optoelectronic properties. Meanwhile, low-temperature approaches, including solvent engineering and additive incorporation, have facilitated compatibility with flexible and tandem device architectures. Component engineering via A/B/X-site doping has improved structural stability and achieved effective defect passivation. Alkali metal cations and transition metal dopants have played critical roles in reducing phase segregation and suppressing non-radiative recombination. Interface engineering, particularly at the ETL/perovskite and perovskite/HTL interfaces, has effectively reduced energy barriers and significantly enhanced the efficiency of charge extraction. The development of novel CTLs, including inorganic metal oxides, polymers, and organic small molecules, has further elevated device performance, with efficiencies surpassing 18% in optimized systems.

Despite these strides, challenges persist. Achieving uniformly high-quality films with minimal defects, understanding interfacial energetics, and addressing long-term stability under operational conditions remain critical hurdles. Future research should prioritize scalable fabrication methods, advanced interfacial layers, and environmentally stable encapsulation techniques. Additionally, exploring cost-effective, dopant-free charge transport materials and leveraging computational tools for material design will accelerate the commercialization of CsPbI_2_Br PSCs. By addressing these challenges, CsPbI_2_Br-based photovoltaics hold immense potential to complement or surpass existing technologies, paving the way for sustainable and efficient renewable energy solutions.

## Figures and Tables

**Table 1:** CsPbI_2_Br films deposited under high- and low-temperature conditions and the corresponding PV properties of PSCs.

Device Architecture	Active Area (cm^2^)	PCE (%)	J_sc_ (mA/cm^2^)	V_oc_ (V)	FF (%)	Stability	Year^.^	Refs.
FTO/c-TiO_2_/CsPbI_2_Br/Spiro-OMeTAD/Au	0.07	10.7	12.0	1.23	73	Remained stable for a long period in a humid atmosphere	2017	[36]
FTO/c-TiO_2_/CsPbI_2_Br/Spiro-OMeTAD/Au	/	10.56	13.61	1.13	68.64	Exhibited thermal stability under 100 °C annealing for more than a week	2018	[47]
PET/ITO/Nb_2_O_5_/CsPbI_2_Br/ Spiro-OMeTAD/Au	/	11.7	14.6	1.19	67.3	Maintained 70% of their original PCE without any encapsulation after being stored in ambient air for 700 h	2018	[41]
ITO/PEDOT/CsPbI_2_Br/C_60_/BCP/Ag	0.0485	8.67	12.4	1.16	60.1	Showed improved humidity stability	2018	[48]
ITO/NiO_x_/CsPbI_2_Br/C_60_/BCP/Ag	0.1	10.4	12.6	1.05	78.7	Maintained 70% of their initial values	2018	[40]
FTO/TiO_2_/Al_2_O_3_/CsPbI_2_Br/NiO_x_/carbon	/	8.44	14.33	0.945	62.5	Retained its color all the time, even after storage in ambient air (25 °C, 30% RH) for 36 h	2019	[42]
ITO/SnO_2_/CsPbI_2_Br/PTAA/MoO_3_/Al	0.16	13.8	15.58	1.19	74.1	/	2019	[49]
ITO/SnO_2_/CsPbI_2_Br/carbon	0.04	10.44	14.25	1.14	64.12	Displayed only ~10% PCE loss after both 1000 h of storage in N_2_ and continuous heating at 85 °C after 300 h in a N_2_-filled glove box	2019	[50]
FTO/SnO_2_/CsPbI_2_Br/Spiro-OMeTAD/Au	/	11.68	16.01	1.11	65.7	Maintained its efficiency of 50% after 10 d	2019	[44]
ITO/c-TiO_2_/CsPbI_2_Br/Spiro-OMeTAD/Au	0.1	16.07	16.79	1.23	77.81	Retained 95% of its initial PCE after 1000 h in an air atmosphere with an RH of 30%	2019	[51]
ITO/NiO_x_/CsPbI_2_Br/PCBM/Ag	0.04	12.32	15.79	1.02	76.68	Retained over 95% of its initial PCE after being stored in a N_2_ glove box for over 1000 h	2020	[52]
ITO/SnO_2_/CsPbI_2_Br/P3HT/Au	0.1	15.50	14.75	1.231	85.37	Maintained a PCE of more than 95% of the initial value after being stored in an environment with humidity below 25% for 1300 h	2020	[53]
FTO/MoO_3_/CsPbI_2_Br/carbon	0.09	14.84	16.62	1.207	73.83	Retained its initial original color after being kept in ambient air with a specified RH of 20–25% and at room temperature for 9 h	2021	[37]
FTO/TiO_2_/PVP-CsPbI_2_Br/Spiro-OMeTAD/carbon	0.06	10.47	18.47	1.01	56.35	Maintained a stable cubic phase at ambient temperatures for 4 d	2022	[54]
ITO/bi-SnO_2_/CsPbI_2_Br/P3HT/Au	0.16	17	16.6	1.27	80.5	Had excellent thermal stability at 300 °C	2022	[45]
FTO/c-TiO_2_/CsPbI_2_Br/carbon	0.045	15.57	16.74	1.161	80.2	Retained 91.7% of the initial PCE	2023	[38]
FTO/SnO_2_/CsPbI_2_Br/Spiro-OMeTAD/Au	/	16.44	15.76	1.31	79.63	Maintained more than 88% of the initial PCE after 1000 h	2024	[46]

**Table 3:** A/B/X-site doping engineering of CsPbI_2_Br films and the PV performance of the corresponding PSCs.

Device Architecture	Active Area (cm^2^)	PCE (%)	J_sc_ (mA/cm^2^)	V_oc_ (V)	FF (%)	Stability	Year	Refs.
FTO/TiO_2_/Cs_0.925_K_0.075_PbI_2_Br/Spiro-OMeTAD/Au	0.15	10.0	11.6	1.18	73	Maintained 80% of its initial value for 120 h at 20 °C and with RH = 20%	2017	[27]
FTO/c-TiO_2_/mp-TiO_2_/CsPb_0.98_Sr_0.02_I_2_Br/P3HT/Au	0.159	11.3	14.9	1.067	70.9	Remained stable for 3 weeks, with its PCE rising during the first week, under storage conditions of 25 °C and RH < 50% in the dark	2017	[71]
FTO/NiO_x_/InCl_3_:CsPbI_2_Br/ZnO@C_60_/Ag	0.09	13.57	15.1	1.15	78	Maintained its initial PCE for over 80 h in a sealed container with an RH of ~30%	2018	[26]
FTO/c-TiO_2_/CsPb_0.9_Zn_0.1_I_2_Br/Spiro-OMeTAD/Ag	0.07	13.6	15.8	1.18	72.7	Maintained 85% of its initial performance after 400 h in air (25 °C and RH of 20%)	2019	[72]
FTO/c-TiO_2_/m-TiO_2_/CsPb_0.8_Ba_0.2_I_2_Br/N,N-di-p-methoxyphenyl-amine/Spiro-OMeTAD/Au	0.16	14.0	14.0	1.28	78.2	Maintained ~80% of the initial efficiency after 450 h	2019	[73]
FTO/TiO_2_/Eu(Ac)_3_:CsPbI_2_Br/Spiro-OMeTAD/Au	0.09	15.25	15.44	1.25	79.00	Retained 82% of its initial value after 30 d of aging at 25 °C and 35–40% RH	2019	[74]
FTO/TiO_2_/CsPb_0.95_Eu_0.05_I_2_Br/Spiro-OMeTAD/Au	0.16	13.71	14.63	1.223	76.6	Retained 93% of the initial efficiency after 370 h	2019	[75]
ITO/SnO_2_/CsPb_0.995_Nb_0.005_I_2_Br/Carbon	0.08	10.42	12.06	1.20	72	Film has no changes even after 96 h	2019	[76]
FTO/c-TiO_2_/m-TiO_2_/CsPb_0.995_Nb_0.005_I_2_Br/P3HT/Au	0.09	16.45	16.23	1.317	77	Maintained 90% of its initial PCE after 96 h of storage in ambient conditions at 30% RH	2020	[77]
FTO/TiO_2_/Cs_0.99_Rb_0.01_PbI_2_Br/P3HT/Au	0.09	17.16	16.25	1.320	80.03	Maintained >90% initial efficiency in ambient conditions without encapsulation for 120 h with RH < 40%	2020	[68]
FTO/SnO_2_/Cs_0.9_Rb_0.1_PbI_2_Br/GABr/Spiro-OMeTAD/Au	0.06	15.6	15.9	1.25	78.5	Retained 88% of its initial PCE after 60 d of storage in an ambient atmosphere at 25 °C with 25% RH	2020	[69]
FTO/TiO_2_/Cs_0.995_Na_0.005_PbI_2_Br/Carbon	0.125	14.63	14.13	1.267	80.66	Maintained 90% of its initial value under an ambient environment with an RH of 40–60% after 330 h	2021	[64]
FTO/SnO_2_/CsPb_0.995_Fe_0.005_I_2_Br/PCBM/Au	0.16	17.1	15.90	1.31	81.8	A retention of over 95% of the initial PCE after 330 h of MPP tracking	2021	[78]
FTO/c-TiO_2_/m-TiO_2_/CsPb_0.98_Sr_0.02_I_2_Br/P3HT/Au	0.09	14.57	15.19	1.309	73.30	Maintained >85% of its initial PCE in ambient conditions for 100 h at 35% RH	2021	[79]
ITO/SnO_2_/NaCl + N-GQDs:CsPbI_2_Br/P3HT/Ag	/	15.37	15.89	1.24	78	Maintained about 96% of the initial PCE after being stored in air for 270 h under 20–30% RH	2022	[80]
FTO/c-TiO_2_/m-TiO_2_/CsPb_0.95_Ba_0.05_I_2_Br/P3HT/Au	0.09	14.07	15.53	1.202	75.38	/	2023	[66]
FTO/NiO_x_/CsBr:CsPbI_2_Br/PCBM/MgF/Ag	/	15.6	15.5	1.27	79	Retained more than 80% of its initial efficiency after 240 h of continuous illumination	2023	[81]
FTO/c-TiO_2_/Cs_0.7_FA_0.3_PbI_2_Br/Spiro-OMeTAD/Au	0.058	14.55	16.3	1.2	74.1	Maintained 98% of the initial PCE after 1000 s irradiation at AM 1.5G	2024	[70]
ITO/SnO_2_/CaInCsPbI_2_Br/Spiro-OMeTAD/Au	0.1	16.60	16.09	1.29	80.02	Maintained 90% PCE after aging for 2400 h in ambient air with an RH of 50 ± 5%	2024	[82]
ITO/ZnO/CsPb_0.996_Zr_0.004_I_2_Br/Spiro-OMeTAD/Ag	/	16.60	15.84	1.29	81.14	Retained over 91% of the initial PCE after 1000 h of aging in ambient air conditions	2024	[83]

**Table 4:** CsPbI_2_Br dopant strategies and the PV performance of the corresponding PSCs.

Device Architecture	Active Area (cm^2^)	PCE (%)	J_sc_ (mA/cm^2^)	V_oc_ (V)	FF (%)	Stability	Year	Refs.
ITO/c-TiO_2_/CsPbI_2_Br + PTFE/PBDB-T/MoO_3_/Ag	0.10	16.40	16.16	1.24	82.05	Retained 91% of its initial PCE value after 500 h in an ambient atmosphere with an RH of ~30%	2021	[95]
ITO/SnO_2_/LiAc:CsPbI_2_Br/PCBM/Ag	0.04	16.05	16.21	1.30	76.2	Almost no PCE degradation after over 300 h of thermal aging at 85 °C	2022	[96]
FTO/SnO_2_/CsPbI_2_Br@FAAc/Spiro-OMeTAD/Ag	0.09	15.86	16.41	1.22	77	/	2022	[97]
ITO/SnO_2_/ZnO/CsPbI_2_Br:DIM/P3HT/Au	0.04	16.42	16.11	1.236	82.47	Maintained about 85% of the initial PCE after aging at 85 °C for 360 h	2022	[98]
FTO/NiO/Perovskite + ILs/PCBM/BCP/Au	/	19.8	23.8	1.08	81.0	/	2019	[99]
ITO/SnO_2_/CsPbI_2_Br + CsPbBr_3_ NCs/CsPbBr_3_ NCs/P3HT/Ag	/	17.03	15.84	1.37	78	/	2023	[100]
ITO/SnO_2_/CsPbI_2_Br + SiO_2_/P3HT/Ag	/	15.32	16.21	1.23	77	Maintained ~83% of the initial PCE in air with an RH of 20–30% after 160 h of storage	2023	[101]
ITO/SnO_2_/ZnO/CsPbI_2_Br + PEAI/P3HT/Au	0.04	17.40	16.47	1.264	83.56	Retained ~87.25% of its initial PCE at 85 °C in a dry box for 720 h without encapsulation	2023	[102]
FTO/c-TiO_2_/m-TiO_2_/PTACl-CsPbI_2_Br + GAI/P3HT/Au	0.09	16.88	16.25	1.34	77.75	Maintained over 95% of its initial efficiency under continuous illumination with a 100 mW/cm^2^ LED white light for more than 1000 h	2023	[103]
FTO/c-TiO_2_/m-TiO_2_/(2D/3D CsPb_2_I_4_Br/CsPbI_2_Br BHJ) + CsPbI_2_Br/Carbon	0.125	15.25	14.60	1.32	79.11	Maintained 97% of its initial efficiency after being stored for 1250 h at room temperature	2023	[104]
FTO/m-TiO_2_/ErCl_3_:CsPbI_2_Br/PCBM/Ag	0.09	16.74	16.16	1.304	79.44	Maintained 90% of the initial PCE after 400 h at 65 °C and ~30% RH	2023	[105]
ITO/SnO_2_/K-TFA:CsPbI_2_Br/P3HT/Au	0.24	17.1	15.11	1.382	82.0	Retained over 92% of its initial efficiency after aging for 1500 h under ambient air	2023	[106]
ITO/SnO_2_/KAc:CsPbI_2_Br/PCBM/Ag	0.04	15.10	15.84	1.23	77.5	Maintained 88% of its initial value after aging for over 400 h under conditions of 85 °C in a N_2_-filled glove box	2023	[107]
FTO/SnO_2_/NaFo:CsPbI_2_Br/P3HT/Au	0.096	17.66	15.25	1.337	84.52	Retained almost 97% of its initial PCE for 1000 h under 10% RH at room temperature without any encapsulation	2023	[108]
ITO/Spiro-OMeTAD@PTAA/5′-CMP:CsPbI_2_Br/PC_61_BM/BCP/Ag	0.04	15.94	16.58	1.17	82.20	Retained 95% of the PCE after 600 h in air with an RH of 25 ± 5%	2023	[109]
FTO/SnO_2_/DAD:CsPbI_2_Br/Spiro-OMeTAD/Au	0.09	17.38	15.87	1.31	83.60	Retained 90% of its initial efficiency after aging for 1000 h in air with an RH ranging from 20% to 30%	2023	[110]
FTO/SnO_2_/CsPbI_2_Br + AA + HBC/Carbon	0.08	12.71	14.50	1.22	72	Maintained 93% of the initial efficiency after 20 d in air with an RH ranging from 20% to 30%	2023	[111]
FTO/c-TiO_2_/CsPbI_2_Br + OA/Carbon	0.045	15.57	16.74	1.161	80.2	Retained 91.7% of the initial PCE in dry conditions	2023	[38]
ITO/SnO_2_/ZnO_2_/CsPbI_2_Br + PLA/PTAA/MoO_3_/Ag	0.04	18.06	15.89	1.393	81.55	Retained 92% of its initial PCE after being stored in air with an RH of 15–20% for 62 d	2023	[112]
FTO/c-TiO_2_/m-TiO_2_/CsPbI_2_Br + PTM/Carbon	0.09	13.95	15.78	1.30	68	Maintained 91% of its initial efficiency after being stored in ambient air for 14 d	2023	[113]
ITO/SnO_2_/CsPbI_2_Br + (2D)SnSe nanosheets/P3HT/Ag	0.15	14.24	16.02	1.22	72.46	/	2024	[114]
ITO/ZnO/PbAc_2_:CsPbI_2_Br/P3HT/Au	0.09	16.19	16.16	1.26	79.38	Maintained 96.7% of its initial PCE for 1500 h at room temperature and ~25% RH	2024	[115]
ITO/SnO_2_/MAAc:CsPbI_2_Br/P3HT/Au	0.1/1	18.14/16.2	15.54/15.5	1.40/1.34	83.4/79.0	Maintained 95% of its initial efficiency after heating at 85 °C for 1000 h	2024	[116]
FTO/SnO_2_/BP-9:CsPbI_2_Br/Spiro-OMeTAD/Au	/	17.11	15.82	1.34	80.71	Maintained more than 95% of its initial PCE after 1000 h under the conditions of 25 °C and ~50% RH	2024	[117]
FTO/SnO_2_/CsPbI_2_Br + 2,5-TDCA/Carbon	0.1	13.42	14.95	1.31	68.4	Maintained more than 80% of the initial PCE after 240 h under 85 °C and an RH of 20–30%	2024	[118]
FTO/SnO_2_/SnCl_2_/CsPbI_2_Br-PGMA/Spiro-OMeTAD/Au	0.09	10.29	18.64	1.22	45	Retained ~80% of its initial PCE after being stored in air at 25 °C with an RH of 50% for 600 h	2024	[119]
ITO/SnO_2_/CsPbI_2_Br + ABS/Carbon	/	14.27	14.69	1.300	74.7	Retained ~86% of the PCE after being exposed to air with an RH of ~40% for 150 h	2024	[120]
FTO/c-TiO_2_/CsPbI_2_Br + IIA/Carbon	0.09	14.85	14.713	1.306	77.32	Reached 70% after 300 h in air with an RH of 10%	2024	[121]
FTO/c-TiO_2_/CsPbI_2_Br + phenyl-amide/Carbon	0.09	15.51	14.758	1.300	80.85	Retained 91.5% of the initial efficiency after being stored in the dark for 1000 h at 25 °C and an RH of 10%	2024	[122]
ITO/SnO_2_/CsPbI_2_Br + DHBP/P3HT/Ag	0.104	16.86	15.28	1.38	80.05	Retained 95% of the initial PCE during 110 min of aging under continuous heating at 85 °C	2024	[123]
FTO/SnO_2_/CsPbI_2_Br + DBM/Carbon	0.09	13.46	15.26	1.189	74.2	Retained 90% of its initial value with an RH of 15–25% for 500 h	2024	[124]
FTO/c-TiO_2_/CsPbI_2_Br + cytosine/Carbon	0.04	15.00	14.721	1.317	77.34	Retained 94.74% of its initial efficiency after being stored for 65 d under the condition of 10% RH in the air and a temperature of 25 °C	2024	[125]
ITO/SnO_2_/ZnO/CsPbI_2_Br + ANT/P3HT/Au	0.04	17.13	16.07	1.278	83.41	Retained ~90% of its initial PCE after 720 h at 85 °C	2024	[126]

**Table 5:** Interface engineering and the PV performance of the corresponding PSCs.

Device Architecture	Active Area (cm^2^)	PCE (%)	J_sc_ (mA/cm^2^)	V_oc_ (V)	FF (%)	Stability	Year	Refs.
FTO/TiO_2_/TiCl_4_: TiCl_3_/CsPbI_2_Br/Carbon	0.125	14.46	14.21	1.28	79.4	/	2021	[128]
ITO/c-TiO_2_/TiO_2_ NaPAs/CsPbI_2_Br/P3HT/MoO_3_/Ag	/	11.35	15.18	1.10	68.8	Retained 60% of the initial PCE after 10 d of aging	2021	[129]
ITO/TiO_2_/BQD/CsPbI_2_Br/PTAA/Au	0.09	15.31	15.18	1.28	78.5	Retained 94% of the initial PCE in ambient air at 20% RH for 40 d	2021	[130]
ITO/ZnO/SnO_2_/CsPbI_2_Br/GABr/Spiro-OMeTAD/Ag	/	16.97	15.90	1.31	81.5	Retained ~85% of its initial PCE in dry air with <20% RH for 960 h	2021	[131]
FTO/c-TiO_2_/CsPbI_2_Br/[PEVIM]Cl/Spiro-OMeTAD/Au	0.1	14.19	15.70	1.16	77.9	Retained 89% of its initial PCE in ambient air with 50% RH for 960 h	2021	[132]
ITO/TiO_2_/CsPbI_2_Br/B-CDs/Spiro-OMeTAD/Au	0.09	16.76	16.01	1.31	80.0	Retained 95.33% of its initial PCE in ambient air with ~25% RH at 30 °C for 1000 h	2021	[133]
ITO/Spiro-OMeTAD/TS-CuPc/CsPbI_2_Br/Nano-Eu_2_O_3_/PC_61_BM/Bphen/Ag	/	14.85	15.53	1.19	80.7	Retained >60% of its initial PCE in ambient air with 60–70% RH for 1200 h	2021	[134]
ITO/NiO_x_/CsPbI_2_Br/BIZI/PCBM/BPhen/Ag	/	14.32	15.59	1.15	79.79	Retained 95% of its initial PCE in ambient air with 15 ± 5% RH at 25 °C for 14 d	2021	[135]
FTO/c-TiO_2_/Ce-doped CsPbI_3_ QD/CsPbI_2_Br/P3HT/Ag	/	16.38	20.55	1.062	74.9	Retained 64% of its initial PCE in a N_2_ glove box for 30 d	2022	[136]
FTO/TiO_2_/CsPbI_2_Br/BMMIMBF_4_/Spiro-OMeTAD/Au	0.09	17.02	15.96	1.33	80.1	Retained 94.4% of its initial PCE in ambient air at ~25% RH for 1440 h	2022	[137]
ITO/NiO_x_/CsPbI_2_Br/BP-HI/ZnO/C_60_/Ag	/	15.36	16.31	1.21	78.2	Retained 85% of its initial PCE in ambient air with ~30% RH at 25 °C for 330 h	2022	[138]
ITO/SnO_2_/ZnO-EAD/CsPbI_2_Br/Carbon	0.09	14.58	14.98	1.27	76.5	Retained 81% of its initial PCE under continuous 80 °C heating in a N_2_ glove box after 24 d	2023	[139]
FTO/SnO_2_/CdCl_2_/CsPbI_2_Br/Carbon	0.0625	14.47	14.30	1.30	77.85	Retained 94% of its initial PCE in ambient air at 15–20% RH for 30 d	2023	[140]
ITO/ZnO-CsTFA/CsPbI_2_Br/Carbon	0.09	14.25	14.95	1.269	75.1	/	2023	[141]
ITO/ZnO/TA/CsPbI_2_Br/PM6/MoO_3_/Ag	0.09	16.56	15.61	1.306	81.22	Retained 90.4% of its initial PCE after 1080 h	2023	[142]
ITO/SnO_2_/CsPbI_2_Br/CsPbBr_3_ NCs/P3HT/Au	/	17.03	15.84	1.37	78.0	/	2023	[100]
FTO/NiO_x_/CsPbI_2_Br/TiO_2_/Al	0.1	17.1	16.4	1.26	83.1	Retained 92% of its initial PCE in ambient air with 80% RH for 1000 h	2023	[143]
ITO/Cu:NiO/IL:CsPbI_2_Br/CsPbBr_3_-QDs/W:Nb_2_O_3_/Ti/Ag	/	15.37	15.31	1.24	81.0	Retained 80% of its nitial PCE in ambient air for 60 h	2023	[144]
ITO/SnO_2_/PS/CsPbI_2_Br/Carbon	/	13.11	14.12	1.25	74.5	Retained 76.6% of its initial PCE after 1000 h in a glove box	2024	[145]
ITO/SnO_2_/NH_4_BF_4_/CsPbI_2_Br/P3HT/Au	/	17.09	14.57	1.43	81.1	Retained 94.5% of its initial PCE after 1000 h of aging at 15 ± 5% RH	2024	[146]
FTO/TiO_2_/Zn(Ac)_2_/CsPbI_2_Br/PCBM/Ag	0.0706	14.20	14.49	1.226	79.95	Retained 86% of its initial PCE after 500 h in dry air	2024	[147]
FTO/TiO_2_/PFPA/CsPbI_2_Br/Carbon	0.1256	14.15	14.41	1.21	79.07	Retained 84.59% of its initial PCE in a N_2_ atmosphere for 30 d	2024	[148]
ITO/NiO_x_/CsPbI_2_Br/CFPMAI/PCBM/BCP/Ag	0.048	14.43	16.31	1.12	79.02	Retained 98.1% of its initial PCE in a N_2_ atmosphere for 984 h	2024	[149]
ITO/NiO_x_/CsPbI_2_Br/TM/PCBM/BCP/Ag	0.048	15.07	16.42	1.16	79.11	Retained 97.07% of its initial PCE in a N_2_ glove box after 1752 h	2024	[150]

**Table 6:** CTL strategies and the PV performance of the corresponding PSCs.

Device Architecture	Active Area (cm^2^)	PCE (%)	J_sc_ (mA/cm^2^)	V_oc_ (V)	FF (%)	Stability	Year	Refs.
ITO/TiO_2_/CsPbI_2_Br/P3HT/Au	0.05	12.02	13.13	1.30	70.4	Retained around 90% of their initial PCE after 960 h of storage in a dry glove box	2018	[18]
FTO/NiMgLiO_x_/CsPbI_2_Br/c- TiO_2_/Bi/Ag	/	14.0	14.72	1.26	76.0	Maintained >90% of their initial PCE at 85 °C in the dark and >92% under continuous illumination at 45 °C for 1000 h	2019	[158]
ITO/SnO_2_/PN4N/CsPbI_2_Br/PDCBT/MoO_3_/Ag	0.04	16.2	15.3	1.30	81.5	High photostability with an efficiency drop of less than 10% under continuous 1-sun-equivalent illumination for 400 h	2019	[159]
FTO/NiO_x_/CsPbI_2_Br/ZnO@C_60_/Ag	0.09	15.19	15.87	1.23	78	Retained ≈86.5% of its initial PCE after being stored for 75 d in a N_2_ atmosphere	2020	[160]
ITO/A-SnO_x_/CsPbI_2_Br/poly (DTSTPD-r-BThTPD)/Au	0.09	15.53	14.25	1.41	77.0	Retained 71.9% of the initial PCE for 900 h	2020	[161]
ITO/Im-SnO_2_/CsPbI_2_Br/Spiro-OMeTAD/Au	0.1	16.10	15.49	1.31	79.13	Retained ~96% of its initial PCE after being kept in an ambient atmosphere with an RH of 30% and 25 °C for 1000 h	2020	[162]
ITO/PEDOT:PSS/AZO/CsPbI_2_Br/t-BCA/PTAA/MoO_3_/Ag	0.1	15.08	15.87	1.26	75.41	Retained 93% of the original PCE after being stored for 60 d	2020	[163]
ITO/SnO_2_/ZnO_2_/CsPbI_2_Br/SY1 or SY2 or SY3/MoO_3_/Ag	0.09	13.41	14.93	1.19	75.5	Dropped to 82% under the same stored conditions	2020	[164]
ITO/P3CT/CsPbI_2_Br/ZnO:TPPPS@C_60_/Ag	/	14.62	15.51	1.228	76.83	Retained ~80% of its initial efficiency for 32 d under illumination	2021	[165]
ITO/TMACl-SnO_2_/CsPbI_2_Br/TFB/P3HT/Au	0.1	13.84	15.29	1.16	77.53	Retained 68% of its initial PCE for 96 h without any encapsulation	2021	[166]
ITO/SnO_2_/CsPbI_2_Br/P3HT/Au	0.0625	17.26	15.86	1.36	80.0	Retained 92% of the initial PCE after 500 h	2021	[167]
ITO/SnO_2_-KF/CsPbI_2_Br/Spiro-OMeTAD/MoO_3_/Ag	0.0625	15.39	14.79	1.31	79.15	Maintained 40% of its initial PCE after 22 d of storage	2021	[168]
ITO/SnO_2_/CsPbI_2_Br/P3HT or SMe-TATPyr/Au	0.1	16.93	14.71	1.38	81.0	Maintained 96% of its initial PCE after aging at 10–25% RH for 1500 h	2021	[169]
ITO/SnO_2_/A-SnO_x_/CsPbI_2_Br/PDTDT/P3HT/Au	0.09	17.36	15.02	1.42	81.3	Kept 88% of the maximum value after 45 d	2021	[170]
ITO/SnO_2_/ZnO/CsPbI_2_Br/PM6/MoO_3_/Ag	0.09	16.06	15.68	1.241	82.54	Maintained ~76% of the initial PCE	2021	[171]
ITO/NiO_x_/CsPbI_2_Br/Ti_0.9_Sn_0.1_O_2_/Ag	0.08	14.0	15.90	1.15	76.6	Retained 98% of the initial efficiency under 85 °C treatment after 65 d	2022	[172]
FTO/NiMgLiO_x_/CsPbI_2_Br/ETLs/Au	0.04	15.04	15.68	1.214	79.0	Maintained 95.2% of its initial PCE after 480 h under continuous light in a N_2_ atmosphere at 45 °C	2022	[173]
ITO/SnO_2_/CsPbI_2_Br/PCDA1/PCDA2/Au	0.055	11.01	14.55	1.07	69.0	Retained 86% of the initial PCE	2022	[174]
ITO/SnO_2_/CsPbI_2_Br/BDTB/BDMA/MoO_3_/Au	0.04	12.32	15.44	1.12	71.5	Sustained 80% of its PCE after 50 d in ambient air	2022	[175]
ITO/SnO_2_/CsPbI_2_Br/PTQ10/P3HT/MoO_3_/Ag	0.048	17.8	15.24	1.40	83.2	Retained 98.3% of its initial value after aging in a glove box for 1112 h	2022	[176]
ITO/ZnO:Ca/CsPbI_2_Br/PM6/MoO_3_/Ag	0.075	16.39	15.36	1.292	80.61	Maintained 87% efficiency after 275 h under continuous 100 mW/cm^2^ illumination in a N_2_ atmosphere	2023	[177]
FTO/TiO_2_@Sb_2_S_3_-MPA/CsPbI_2_Br/Carbon	0.08	14.59	14.60	1.29	77.49	Maintained the initial efficiency of ~96.4% after continuous heating at 85 °C for 28 d under N_2_	2023	[178]
ITO/SnO_2_/CsPbI_2_Br/P3HT/Au	0.0625	16.22	15.68	1.30	80	Retained over 90% of its initial efficiency after 500 h at 85 °C in a N_2_-filled glove box	2023	[179]
ITO/SnO_2_/CsPbI_2_Br/PE51/PE52/PE53/MoO_3_/Ag	0.04	17.65	15.07	1.42	82.48	Maintained about 94% of its initial PCE after being exposed to an air environment with less than 10% RH for 840 h	2023	[180]
ITO/SnO_2_/CsPbI_2_Br/PE61/MoO_3_/Ag	0.04	16.72	14.52	1.40	82.23	Maintained 93% of its initial PCE after aging for 1000 h	2023	[181]
ITO/ZnO/CsPbI_2_Br/D18-Cl/MoO_3_/Ag	0.04	16.73	15.57	1.259	85.34	Maintained 81% of its initial PCE after aging for 1500 h at 85 °C in a N_2_ atmosphere	2023	[182]
ITO/SnO_2_-SA/CsPbI_2_Br/P3HT/Ag	0.0625	16.90	15.90	1.24	0.837	Retained 85% of its initial performance after 700 h of continuous operation under simulated one-sun illumination	2024	[183]
ITO/Spiro-OMeTAD:PTAA/CsPbI_2_Br/FADD:ZnO/C_60_/Ag	0.04	16.05	16.19	1.222	81.14	Maintained 80% of its original PCE after continuous heating for 1200 h	2024	[184]
